# Regulation of PERK expression by FOXO3: a vulnerability of drug-resistant cancer cells

**DOI:** 10.1038/s41388-019-0890-7

**Published:** 2019-07-16

**Authors:** Glowi Alasiri, Yannasittha Jiramongkol, Stefania Zona, Lavender Y. -N. Fan, Zimam Mahmud, Gyungyub Gong, Hee Jin Lee, Eric W. -F. Lam

**Affiliations:** 1 Department of Surgery and Cancer, Imperial College London, Hammersmith Hospital Campus, London, W12 0NN UK; 2 Department of Pathology, Asan Medical Center, University of Ulsan College of Medicine, Songpa-gu, Seoul Korea

**Keywords:** Cell biology, Predictive markers, Breast cancer

## Abstract

The major impediment to effective cancer therapy has been the development of drug resistance. The tumour suppressive transcription factor FOXO3 promotes cell cycle arrest, senescence and cell death, and mediates the cytotoxic and cytostatic functions of cancer therapeutics. In consequence, FOXO3 is often downregulated as an adaptive response in cancer and particularly in chemotherapeutic drug-resistant cells. Consistently, we find that FOXO3 expression is attenuated in the drug-resistant MCF-7-Epi^R^ and MCF-7-Tax^R^ compared to the parental MCF-7 breast cancer cells. Using ChIP, short-interfering RNA (siRNA) knockdown, and overexpression assays as well as *Foxo*^*1/3/4*−/−^ MEFs, we establish the endoplasmic reticulum (ER)-stress defence modulator PERK (eIF2AK3) as a direct downstream transcriptional target of FOXO3. In agreement, there is also a positive correlation between FOXO3 and PERK expression at the protein and RNA levels in breast cancer patient samples. We uncover that PERK expression is downregulated but its activity constitutively elevated in the drug-resistant cells. With this in mind, we exploit this adaptive response of low FOXO3 and PERK expression, and high PERK activity in drug-resistant breast cancer cells and show that these drug-resistant cells are specifically sensitive to PERK inhibition. In support of this finding, we show that ectopic overexpression of FOXO3 can reduce the sensitivity of the resistant cells to the PERK inhibitor GSK2606414, while the *Foxo*^*1/3/4*−/−^ MEFs expressing lower levels of PERK are more sensitive to PERK inhibition compared to wild-type MEFs. PERK inhibitor-titration and -time course experiments showed that the drug-resistant cells, which express lower expression and higher activity levels of PERK, are more sensitive to the increasing concentrations of PERK inhibitor compared to parental MCF-7 cells. Our present work thus reveals a chemotherapeutic drug-resistant cancer cell vulnerability in PERK and suggests PERK as a potential target for cancer therapy, specifically in the context of drug-resistant cancers.

## Introduction

Cells orchestrate a finely tuned balance between protein synthesis and degradation to maintain protein homoeostasis (*proteostasis*). Endoplasmic reticulum (ER) is a cellular organelle with a central role in maintaining proteostasis through its involvement in protein synthesis, folding, quality control and distribution. Defective proteostasis leads to the accumulation of misfolded or unfolded proteins in the ER lumen, which will cause ‘ER stress’. This cellular stress condition triggers the unfolded protein response (UPR), a highly conserved reaction, which aims to restore proteostasis. However, this survival-promoting function of the UPR can switch to a cell death-inducing mode if proteostasis is overwhelming and cannot be rectified in time. UPR is activated upon the detection of the misfolded/unfolded protein accumulation in the ER by the Heat Shock 70 kDa Protein 5 (HSPA5) also known as glucose-regulated protein 78 (GRP78). UPR is mediated primarily through three signalling axes driven by three ER-transmembrane signalling proteins, IRE1α (Inositol Requiring 1α), PERK (PKR-like ER Kinase) and ATF6α (activating transcription factor 6α), respectively.

Cancer cells are exposed continuously to external and intrinsic factors that alter protein homoeostasis, resulting in heightened ER stress. Point mutations, large scale gains and losses of genomic materials, and hyperactive protein synthesis pathways challenge this well-poised balance in cancer cells [1, 2]. As a consequence, cancer cells devise adaptive UPR to promote viability and to enhance the protein folding and clearance capacity to restore proteostasis. It has also been proposed that cancer cells can modulate UPR to acquire competitiveness and survival advantages to drive cancer initiation, progression and drug resistance. While the pro-survival side of the UPR underlies its ability to sustain and promote cancers, its cell death-inducing functions can be exploited for cancer therapy. In consequence, a better understanding of the role and regulation of these ER stress regulators might allow us to devise new therapeutic strategies to interrupt ER stress signalling, which specifically target cancer and drug-resistant cells. Currently, a number of ER stress pathway inhibitors have been developed and they are being evaluated in preclinical studies and in early clinical trials [3]. For example, in the clinic, the most advanced inhibitors are rapamycin analogues, such as temsirolimus and everolimus, that selectively inhibit mTORC1 and have received approval for the treatment of advanced renal cell carcinoma [4]. In addition, a selective first in-class PERK inhibitor, GSK2606414, which restricts PERK activity has been demonstrated in mouse xenograft models with efficacies in limiting human tumour growth [5].

The Forkhead box (O3) transcription factor plays a critical role in promoting cell cycle arrest, senescence and cell death, as well as mediating the cytotoxic and cytostatic functions of cancer therapeutics. FOXO3 acts downstream of the phosphatidylinositol 3-kinase-protein kinase B (PI3K-PKB/AKT) signalling pathway as a tumour suppressor, preventing the transmission of potentially oncogenic mutations and activities by negatively controlling cellular proliferation. For example, FOXO3 regulates the expression of negative cell proliferation regulators, such as p27^Kip1^ and BIM [6–9] but represses the expression of oncogenes, including FOXM1 [6, 10, 11]. Like other tumour suppressors, FOXO3 is frequently downregulated or inactivated in cancer and particularly, in drug-resistant cells, often as a result of hyperactive PI3K-AKT signalling [6, 12]. Two other mammalian FOXO family members, FOXO1 and FOXO4, which bear high levels of homology with FOXO3 and regulate multiple common target genes, giving rise to functional redundancy between FOXO proteins [13]. Moreover, FOXO3 also regulates the expression of FOXO1, -3 and -4 in a positive feed forward mechanism [14]. PERK has previously been shown to phosphorylate FOXO3 in an AKT-independent manner to promote FOXO3 nuclear relocation and activation [15–17].

ER defences have been implicated in chemoresistance [18]. Targeted suppression of ER defences has been shown to improve therapeutic outcomes of multidrug-resistant cancer cells [19, 20]. Here, we identify the ER-stress modulator PERK as a direct downstream target of FOXO3 and explore the regulation of PERK expression by FOXO3 as a vulnerability in drug-resistant cancer cells.

## Results

### PERK expression correlates positively with FOXO3 activity in response to drug treatment

**Fig. 1:**
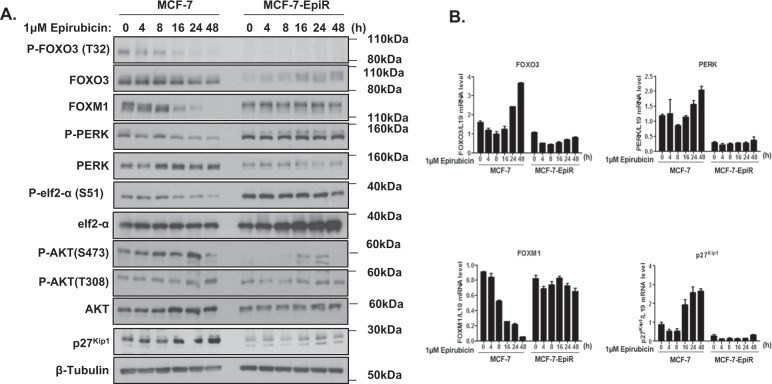
Correlations between PERK and FOXO3 expression and activity in MCF-7 and MCF-7-Epi^R^ cells in response to epirubicin. **a** The epirubicin-sensitive MCF-7 and -resistant MCF-7-Epi^R^ cells were either left untreated or treated with 1 µM epirubicin for the times shown. Whole-cell protein lysates were then analysed by western blotting using the antibodies against the proteins indicated. Molecular weight markers are shown. The protein expression levels of P-FOXO3 (T32) (95 kDa), FOXO3 (95 kDa), FOXM1 (110 kDa) and ER stress molecules, including P-PERK (T981) (140 kDa), PERK (140 kDa), P-eIF2a (S51) (38 kDa), eIF2a (38 kDa), P-AKT (S473) (60 kDa), P-AKT (T308) (60 kDa), AKT (60 kDa), p27^Kip1^ (27 kDa) and β-Tubulin (55 kDa) were investigated. **b** FOXO3, PERK, FOXM1, p27^Kip1^ mRNA expression after treatment with epirubicin as determined by RT-qPCR. Representative RNA expression profiles of at least three independent experiments. Three technical repeats were conducted in one experiment, and the data were normalised to L19 and displayed as means ± S.E.M. The expression trends of mRNA species between MCF-7 and MCF-7-Epi^R^ cells are compared using two-way ANOVA (Significant ***p < 0.001, for all mRNA species, respectively)

PERK is a key ER stress sensor that triggers adaptive signalling programmes to restore proteostasis and has been shown to directly phosphorylate FOXO3 to promote its nuclear relocalization and activity [15–17]. To investigate a potential role of PERK in modulating cytotoxic drug response and resistance, we studied the expression of PERK and its putative substrates FOXO3 and eIF2α in the drug-sensitive MCF-7 and the -resistant MCF-7-Epi^R^ cells. Western blot analysis showed that P-PERK (T981) levels, which reflect its activity, were low in MCF-7 but high in the resistant MCF-7Epi^R^ cells (Fig. 1a). Conversely, the expression levels of FOXO3 and its downstream transcriptional target p27^Kip1^ were low in the resistant cells but were comparatively much higher in MCF-7 cells, consistent with the role of FOXO3 as a tumour suppressor (Fig. 1a). In agreement with previous findings, epirubicin induced dephosphorylation/activation of FOXO3 and led to upregulation of FOXO3 targets, including p27^Kip1^, in MCF-7 and not MCF-7-Epi^R^ cells [21–24]. In concordance, epirubicin also caused the downregulation of FOXM1, a potent oncogene repressed by FOXO3 [11] (Fig. 1a). Interestingly, PERK mRNA and protein levels were induced by epirubicin in a similar manner as the p27^Kip1^ and FOXO3 mRNA transcripts in MCF-7 cells, but remained constitutively low in MCF-7-Epi^R^ cells where FOXO3 expression is low (Fig. 1a, b). We also observed that the expression levels of FOXO3 mRNA correlated directly with FOXO3 dephosphorylation, consistent with previous findings that FOXO3 regulates FOXO gene expression [14]. We also found that like p27^Kip1^ and FOXO3, the kinetics of PERK mRNA expression also mirrored that of FOXO3 dephosphorylation in response to epirubicin, suggesting that FOXO3 regulates PERK expression at the transcription level (Fig. 1b). Collectively, these data led us to hypothesise that cytotoxic agents can activate FOXO3 through downregulating PERK activity and that PERK might be a downstream transcriptional target of FOXO3. We also observed that the expression of P-PERK (T981) and P-FOXO3 (T32) decreased in MCF-7 upon epirubicin treatment, while the total PERK and FOXO3 proteins remained relatively constant, suggesting that epirubicin regulates PERK and FOXO3 activity. While PERK has been shown to be able to phosphorylate and activate FOXO3 directly [25], our findings suggest that the dephosphorylation and activation of FOXO3 by epirubicin is associated with PERK inactivation. Notably, upon epirubicin treatment, PERK and FOXO3 became dephosphorylated in a similar manner, suggesting that epirubicin inhibits PERK activity and that the downregulation of PERK activity by epirubicin mediates FOXO3 dephosphorylation (T32)/activation. Notably, western blot and reverse transcription quantitative polymerase chain reaction (RT-qPCR) analyses also revealed similar results in MCF-7 and MCF-7-Tax^R^ cells in response to paclitaxel treatment (Supplementary Fig. S1).

### FOXO3 directly regulates PERK expression

**Fig. 2:**
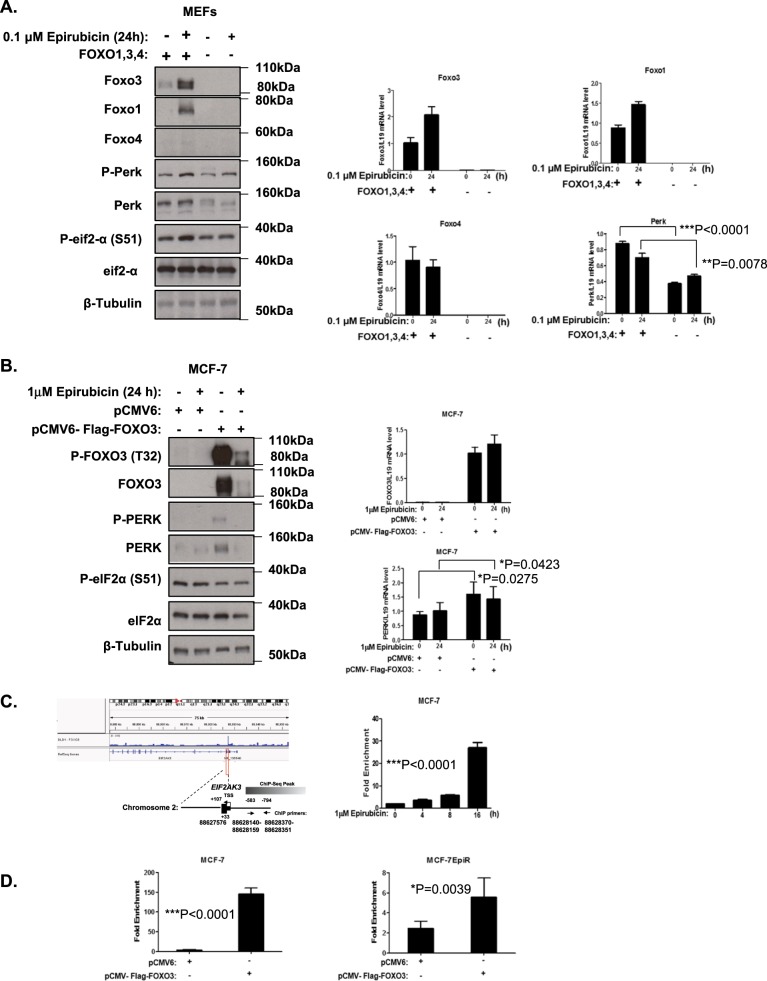
FOXO3 regulates PERK expression in MCF-7 and MEFs. **a** Expression levels of protein and mRNA in WT and *Foxo*^*1/3/4*−/−^ MEFs in response to epirubicin. Western blotting was performed to determine the protein expression levels for P-Foxo3 (T32) (95 kDa), Foxo3 (85 kDa), P-Foxo1 (T24) (79 kDa), Foxo1 (79 kDa), Foxo4 (54 kDa), P-Perk (T981) (140 kDa), Perk (140 kDa), P-eIF2a (S51) (38 kDa), eIF2a (38 kDa) and β-Tubulin (55 kDa). Foxo3, Foxo1, Foxo4 and Perk mRNA levels were investigated by RT-qPCR. Three technical repeats were conducted in one experiment, and the data were normalised to L19 and displayed as means ± SEM. (*n* = 6; two-tailed *t*-test). Representative RNA expression profiles of at least three independent experiments are shown (right panel). **b** Expression levels of protein and mRNA in MCF-7 cells overexpressing FOXO3. P-FOXO3 (T32) (95 kDa), FOXO3 (85 kDa), P-FOXO1 (T24) (79 kDa), FOXO1 (79 kDa), P-PERK (T981) (140 kDa), PERK (140 kDa), P-eIF2a (S51) (38 kDa), eIF2a (38 kDa) and β-Tubulin (55 kDa) as loading control. FOXO3, FOXO1, FOXO4 and PERK mRNA were determined by RT-qPCR. Three technical repeats were conducted in one experiment, and the data were normalised to L19 and displayed as means ± SEM. (*n* = 6; two-tailed *t*-test). (right panel). **c** FOXO3-binding site on human PERK promoter. ChIP-sequencing data of FOXO3-binding in DLD1 colon carcinoma cells [26] were used for predicting FOXO3-binding sites using the Integrative Genomics Viewer (Version 2.3.88) and the hg19 UCSC Genome Browser 45. The predicted binding profiles of FOXO3, and the locations of the designed ChIP primer pairs are aligned to the human *PERK* promoter. MCF-7 cells treated with 1 μM epirubicin for 0, 4, 8 and 24 h were used for chromatin immunoprecipitation assays using the IgG as negative control and anti-FOXO3 antibody. After reversal of cross-linking, the co-immunoprecipitated DNA was amplified by qRT–PCR, using primers amplifying the FOXO3-binding-site-containing region. Data are displayed as means ± SEM (*n* = 3; one-way ANOVA). **d** ChIP analysis of FOXO3 binding on *PERK* promoter in MCF-7 and MCF-7-Epi^R^ cells transfected with empty vector or FOXO3 expression vector. Representative RNA expression profiles of at least three independent experiments are shown. Three technical repeats were conducted in one experiment, and data were normalised to IgG and displayed as means ± SEM (*n* = 6; two-tailed *t*-test). Significant: *P < 0.05, **P < 0.01, ***P < 0.001

To test the hypothesis that PERK is a downstream transcriptional target of FOXO3, we first used siRNA to deplete the expression of FOXO3 and the related FOXO1, and studied the expression of PERK in MCF-7 and MCF-7-Epi^R^ cells. The results revealed that silencing of either FOXO3, FOXO1 or both together failed to downregulate PERK expression, and it is likely due to functional redundancy rather than compensatory expression between FOXO proteins (Supplementary Fig. S2), a mechanism, which has previously been suggested [14]. To circumvent this functional redundancy between FOXO proteins, we next studied the expression of PERK in the wild-type (WT) and FOXO1/3/4-deficient mouse embryonic fibroblasts (*Foxo*^*1/3/4*−/−^ MEFs) in response to epirubicin treatment (Fig. 2a). The results showed that PERK mRNA and protein levels were significantly lower in the *Foxo*^*1/3/4*−/−^ MEFs when compared to the WT MEFs, providing further evidence that FOXO proteins regulate PERK expression.

To confirm further that FOXO3 regulates PERK expression at the transcriptional level, we next overexpressed FOXO3 and studied the expression of PERK in MCF-7 cells. Consistent with our previous results, we found that overexpression of FOXO3 caused a significant increase in PERK expression at both the mRNA and protein levels (Fig. 2b). Notably, FOXO3 can promote PERK phosphorylation indirectly through inducing its expression. This FOXO3-mediated PERK phosphorylation is likely to be mediated by the endogenous overactive ER stress signalling in these cancer cells. We next investigated if the regulation of PERK by FOXO3 is at the promoter level. To this end, we first investigated if FOXO3 is recruited to *PERK* promoter in a previously published FOXO3 chromatin immunoprecipitation (ChIP)-Seq study in DLD1 colon carcinoma cells [26]. The analysis revealed that FOXO3 binds to the promoter region of PERK gene in the DLD1 colon carcinoma cells (Fig. 2c). A schematic representation of the primers and the FOXO-binding sites with respect to the PERK transcription start site (TSS) is shown in Fig. 2c. ChIP assay was next performed to examine the binding of FOXO3 to the *PERK* promoter region in the MCF-7 and MCF-7-Epi^R^ cell lines, with FOXO3 primers designed to recognise a region –583 and –794 (bp) upstream of PERK gene (Fig. 2c). Enrichment of FOXO3 binding was observed in this upstream region of the PERK promoter region and not in control region, suggesting that FOXO3 can directly regulate PERK expression at the promoter level. (Fig. 2c, d). Consistent with this, the binding of endogenous FOXO3 could be induced by epirubicin treatment in MCF-7 cells (Fig. 2c). Moreover, ectopic expression of FOXO3 significantly increased the recruitment of FOXO3 to the PERK promoter in both MCF-7 and MCF-7-Epi^R^ cells (Fig. 2d). Interestingly, binding of FOXO3 was lower in the resistant MCF-7-Epi^R^ cells compared to MCF-7. Collectively, these data suggest that FOXO3 directly regulates PERK expression at the promoter level, and that the lower FOXO3 expression levels directly contribute to the low PERK expression levels in the resistant MCF-7-Epi^R^ cells. Notably, although the transcript levels of transfected FOXO3 were comparable in both MCF-7 and MCF-7-Epi^R^ cells, the FOXO3 protein expression levels in substantially higher in MCF-7 compared with MCF-7-Epi^R^ cells, suggesting that FOXO3 expression is also modulated at the post-transcriptional in the drug-resistant cells.

### PERK and P-eIF2α correlates with FOXO3 expression in breast cancer samples

**Fig. 3:**
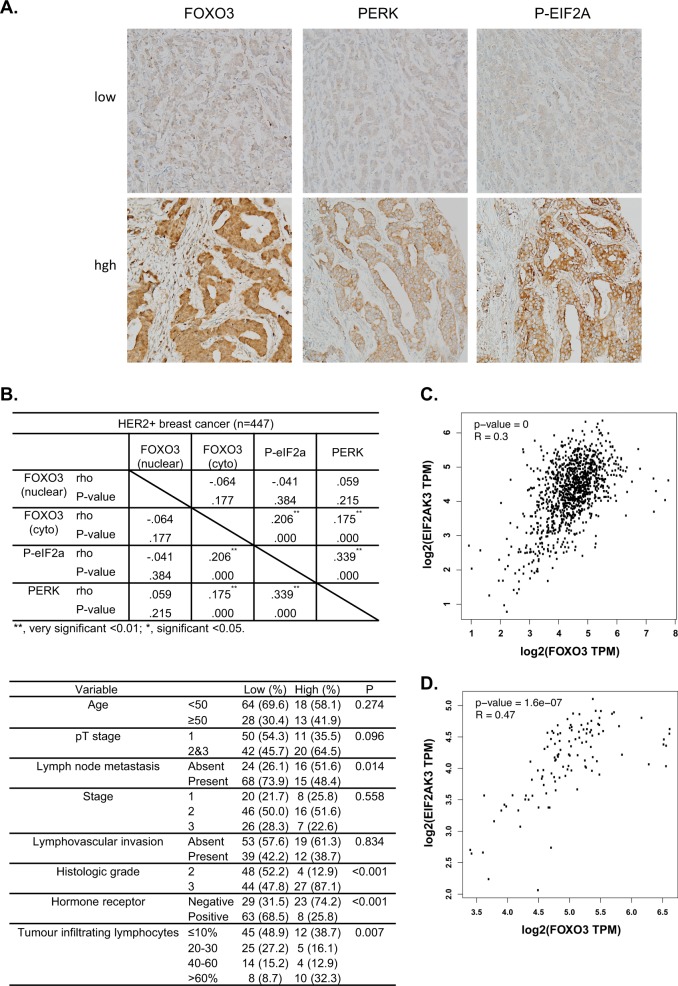
FOXO3 expression correlates with PERK levels in breast cancer patient samples. **a** Representative immunohistochemical staining images from two patient samples (high and low) showing correlations between FOXO3, PERK and P-eIF2a levels in a cohort of HER-2-positive breast cancer patient samples. **b** Staining results and Chi-square analysis. Chi-square statistical analysis was used to test the correlations between FOXO3, PERK and P-eIF2a expression in patients using SPSS 16.0. The correlation of FOXO3 and PERK expression with clinical parameters (lower panel). In statistical analysis, *P < 0.05, **P < 0.01, ***P < 0.001 were considered as significant. **c**, **d** Correlation analysis using Gene Expression Profiling Interactive Analysis (GEPIA) [28]. The results indicated that a strong and positive correlation between FOXO3 and PERK mRNA expression in 1085 breast cancer cases and 291 normal breast tissue samples derived from The Cancer Genome Atlas (TCGA) database, respectively. ****P < 0.0001

To provide further physiological evidence that FOXO3 regulates PERK and to investigate its potential relevance in breast cancer, FOXO3, PERK and P-eIF2α expression was assessed by immunohistochemical (IHC) staining in a HER2-positive cohort of breast cancer patient samples (Fig. 3a). IHC results revealed that cytoplasmic and not nuclear FOXO3 expression is significantly correlated with PERK and P-eIF2α expression (Pearson coefficient *r* = 0.215, ***P < 0.001 and *r* = 0.175, ***P < 0.000, respectively, for cytoplasmic FOXO3; *r* = 0.059, *P* = 0.215 and *r* = −0.041, *P* = 0.384, respectively, for nuclear FOXO3) (Fig. 3b). This further supports our earlier finding that FOXO3 directly regulates PERK expression and activity, which is reflected by P-eIF2α the downstream phosphorylation target of PERK. Moreover, this analysis showed that FOXO3 expression significantly associated with PERK-eIF2α pathway activation, as revealed by P-eIF2α, which is associated with tumour-infiltrating lymphocytes. Notably, the expression cytoplasmic and not nuclear FOXO3 was correlated with PERK and P-eIF2a expression; nevertheless, this is consistent with a previous finding, which shows that constitutively nuclear FOXO3 localisation signifies deregulated FOXO3 function and predicts poor survival [22]. Tumour-infiltrating lymphocytes (TILs) are a good predictive and prognostic biomarker in human epidermal growth factor receptor 2 (HER2)-positive breast cancer, where abundant TILs in the stroma of invasive breast carcinoma is an independent marker for good prognosis. In agreement, cytoplasmic FOXO3 is also a favourable independent prognostic factor in breast cancer [27]. Moreover, correlation analysis using Gene Expression Profiling Interactive Analysis (GEPIA) [28] indicated that a strong and positive correlation between FOXO3 and PERK mRNA expression in 1085 breast cancer cases and 291 normal breast tissue samples derived from The Cancer Genome Atlas (TCGA) database (Fig. 3c, d). Taken together, these results provide strong evidence that FOXO3 regulates PERK expression in human breast cancer.

### Drug-resistant breast cancer cells are particularly vulnerable to PERK inhibition

**Fig. 4:**
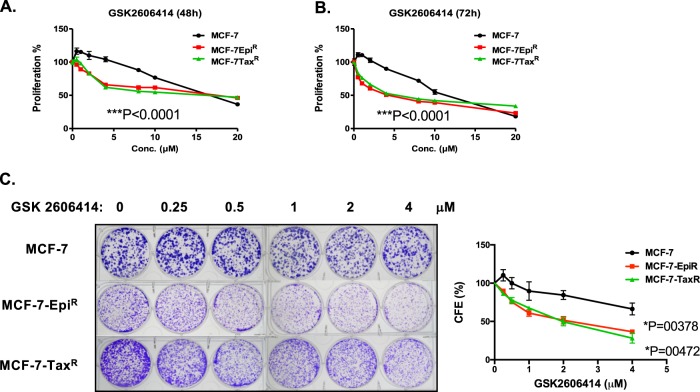
Proliferative and clonogenic assays to examine the ability of MCF-7, MCF-7-Epi^R^ and MCF-7-Tax^R^ to proliferate and form colonies in response to PERK inhibition. **a**, **b** MCF-7, MCF-7-Epi^R^ and MCF-7-Tax^R^ cells were treated with different concentrations of GSK2606414 for 48 and 72 h, respectively, and used for SRB assays. (*n* = 3; one-way ANOVA). **c** MCF-7, MCF-7-Epi^R^ and MCF-7-Tax^R^ cells were treated with 0, 250 nM, 500 nM, 1 mM, 2 mM and 4 mM of PERK inhibitor GSK2606414 every 48 h for 15 days then stained with crystal violet in clonogenic assays. (*n* = 3; one-way ANOVA). Significant *P < 0.05, ***P < 0.001

Our results thus far showed that PERK expression is downregulated but its activity substantially elevated as an adaptive response in the drug-resistant cells (Supplementary Fig. S3). This finding led us to hypothesise that the regulation of PERK by the tumour suppressor FOXO3 is a potential vulnerability of the drug-resistant breast cancer cells. To test this conjecture, we studied the cytotoxic effects of the specific PERK inhibitor GSK2606414 on MCF-7, MCF-7-Epi^R^ and MCF-7-Tax^R^ cells. Sulforhodamine B (SRB) and clonogenic assays revealed that both MCF-7-Epi^R^ and MCF-7-Tax^R^ cells are significantly more sensitivity to GSK2606414 compared to the parental MCF-7 cells (Fig. 4a, b).

### PERK inhibition induces FOXO3 activity via repressing AKT in the drug-resistant cancer cells

**Fig. 5:**
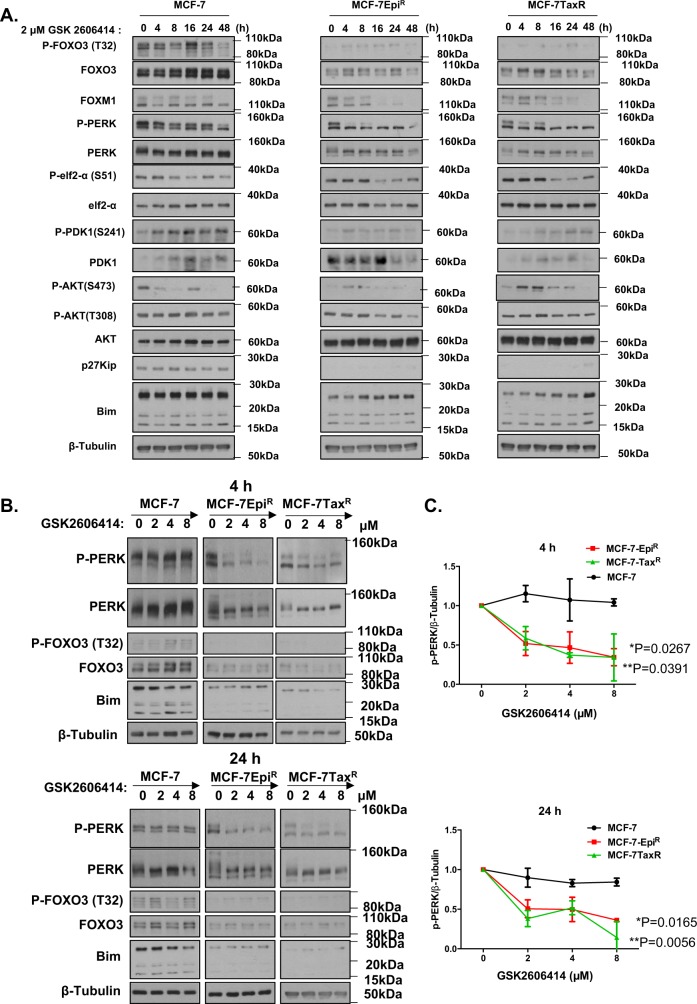
PERK activity in MCF-7, MCF-7Epi^R^ and MCF-7-Tax^R^ cell lines in response to GSK2606414 treatment. **a** Protein expression levels of P-FOXO3 (T32) (95 kDa), FOXO3 (85 kDa), FOXM1 (110 kDa), P-PERK (T981) (140 kDa), PERK (140 kDa), P-eIF2a (S81) (38 kDa), eIF2a (38 kDa), P-PDK1 (S241) (60 kDa), PDK1 (60 kDa) P-AKT (S473) (60 kDa), P-AKT (T308) (60 kDa), AKT (60 kDa), p27^Kip1^ (27 kDa) and Bim (15, 18, 23 kDa) and β-Tubulin (55 kDa) as loading control in MCF-7, MCF-7-Epi^R^ and MCF-7-Tax^R^ cell lines were examined by western blotting after GSK2606414 treatment at different time points indicated. **b** Protein expression levels of P-FOXO3 (T32) (95 kDa), FOXO3 (95 kDa), P-PERK (T981) (140 kDa), PERK (140 kDa), Bim (15, 18, 23 kDa) and β-Tubulin (55 kDa) in MCF-7, MCF-7-Epi^R^ and MCF-7-Tax^R^ cell lines were examined by western blotting with specific antibodies 24 h after treatment with different doses of GSK2606414 as indicated. Representative images are shown. **c** ImageJ densitometry was used to quantify the P-PERK and β-tubulin levels from which independent background readings were subtracted. Western blots are representative of three independent experiments. The relative expression levels shown (right panels) are means ± SEM of the ratios of P-PERK to β-tubulin levels relative to those at 0 h. Data are presented as means ± SEM (*N* = 3) and were analysed by two-way ANOVA. Significant: *P < 0.05, **P < 0.01, ***P < 0.001. ‘ns’ indicates no significant difference

Subsequent western blot results showed that treatment with a high dose (2 μM) of the specific PERK inhibitor GSK2606414 caused a coordinated decline in P-PERK, P-FOXO3 and P-AKT (473) levels, suggesting that PERK inhibition by GSK2606414 induces FOXO3 activation through repressing AKT (Fig. 5a). Notably, the decline in P-PERK, P-FOXO3 and P-AKT (473) was observed after 8 h in both MCF-7-Epi^R^ and MCF-7-Tax^R^ cells, while there were no appreciable changes in P-PERK, P-FOXO3 and P-AKT (473) levels until at least 48 h in MCF-7 cells. The activation of FOXO3 by GSK2606414 in MCF-7-Epi^R^ and MCF-7-Tax^R^ cells was mirrored by the induction of the pro-apoptotic FOXO3 target BIM in the MCF-7-Epi^R^ and MCF-7-Tax^R^ cells but not in the MCF-7 cells. These data further confirmed that PERK negatively regulates FOXO3 activity via AKT and suggested that MCF-7-Epi^R^ and MCF-7-Tax^R^ cells are more sensitive to PERK inhibition compared to MCF-7 cells, because of their lower PERK levels. Interestingly, P-PDK1 and its target P-AKT (308) did not decline following GSK2606414 treatment, suggesting PERK is unlikely to mediate AKT activation and FOXO3 phosphorylation (T32) via PDK1.

### The drug-resistant MCF-7 cancer cells with low PERK levels is more sensitive to GSK2606414 inhibition

To demonstrate further that the drug-resistant cells are more sensitive to GSK2606414 because of their lower PERK levels, we next performed dose-titration experiments on the drug-sensitive and -resistant MCF-7 cells (Fig. 5b). Western blot results at 4 h and 24 h after GSK2606414 treatment demonstrated that despite possessing higher intrinsic activities, PERK is substantially more sensitive to inhibition by GSK2606414 in the resistant MCF-7-Epi^R^ and MCF-7-Tax^R^ cells as revealed by P-PERK dephosphorylation. Altogether, these results suggested that the drug-resistant cells, which present low PERK expression but high activity are more sensitive to GSK2606414.

### FOXO3 overexpression increases PERK expression and GSK2606414 resistance in drug-resistant MCF-7 cells

**Fig. 6:**
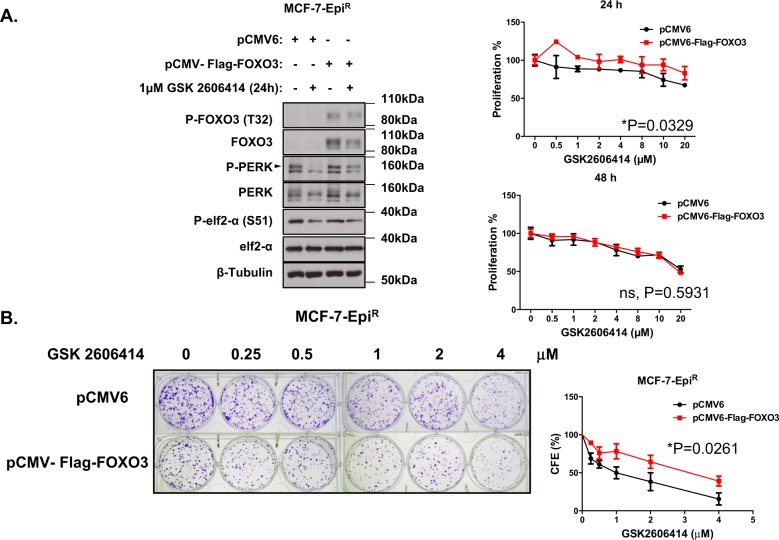
Effects of FOXO3 ectopic expression on MCF-7-Epi^R^ cells in response to GSK2606414. MCF-7-Epi^R^ cells were transfected with empty control or FOXO3 expression vector and left untreated or treated with 2 µM GSK2606414 for 24 h. **a** Western blot to determine the protein expression levels for P-FOXO3 (T32) (95 kDa), FOXO3 (85 kDa) and ER stress molecules, including p-PERK (140 kDa), PERK (140 kDa), P-eIF2a (38 kDa), eIF2a (38 kDa) and β-Tubulin (55 kDa). SRB assays were carried out after 48 h and 72 h after treatment with GSK2606414 at concentrations indicated (right panels). **b** Transfected MCF-7-Epi^R^ cells were treated with 0, 0.25, 0.5, 1, 2 and 4 µM of PERK inhibitor GSK2606414 every 48 h for 15 days then stained with crystal violet in clonogenic assays. Significant *P < 0.05, **P < 0.01, ***P < 0.001

**Fig. 7:**
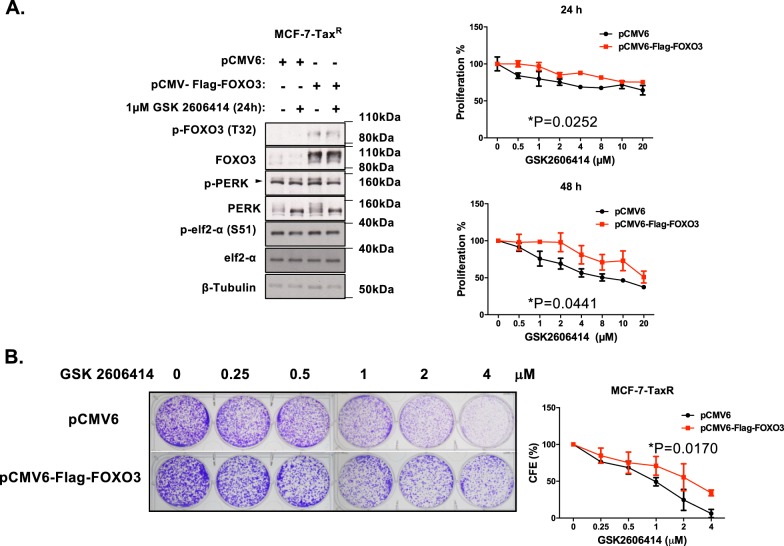
Effects of FOXO3 ectopic expression on MCF-7-Tax^R^ cells in response to GSK2606414. MCF-7-Tax^R^ cells were transfected with empty control or FOXO3 expression vector and left untreated or treated with 2 µM GSK2606414 for 24 h. **a** Western blot to determine the protein expression levels for P-FOXO3 (T32) (95 kDa), FOXO3 (85 kDa) and ER stress molecules, including p-PERK (140 kDa), PERK (140 kDa), P-eIF2a (38 kDa), eIF2a (38 kDa) and β-Tubulin (55 kDa). SRB assays were carried out after 48 h and 72 h after treatment with GSK2606414 at concentrations indicated (right panels). **b** Transfected MCF-7-Tax^R^ cells were treated with 0, 0.25, 0.5, 1, 2 and 4 µM of PERK inhibitor GSK2606414 every 48 h for 15 days then stained with crystal violet in clonogenic assays. Significant *P < 0.05, **P < 0.01, ***P < 0.001

Next, we asked if the regulation of PERK by FOXO3 plays a key part in modulating GSK2606414 sensitivity in the drug-resistant MCF-7 cells (Figs. 6 and 7). To this end, we overexpressed FOXO3 in MCF-7, MCF-7-Epi^R^ and MCF-7-Tax^R^ cells and treated these transfected cells with various doses of GSK2606414. RT-qPCR and western blot analysis showed that FOXO3 ectopic expression induced PERK expression, particularly after GSK2606414 treatment (Figs. 6a and 7a). This provided further evidence that FOXO3 drives PERK expression, while PERK inhibition induces FOXO3 activity. GSK2606414 dose-titration experiments revealed that although FOXO3 ectopic expression lowered the clonogenicity of the MCF-7-Epi^R^ and MCF-7-Tax^R^ cells (Supplementary Fig. S4), it enhanced the resistance of the MCF-7-Epi^R^ and MCF-7-Tax^R^ cells to GSK2606414, suggesting further that the regulation of PERK by FOXO3 plays an integral part in modulating the sensitivity to PERK inhibition (Figs. 6b and 7b). In contrary, FOXO3 overexpression increased the sensitivity of the drug-resistant MCF-7 to the conventional chemotherapeutic drugs, such as MCF-7-Epi^R^ to epirubicin (Supplementary Fig. S5), further confirming FOXO3 has a key role in mediating the cytotoxicity of conventional chemotherapeutics. Consistently, there is little or no effect of FOXO3 ectopic expression on the sensitivity of MCF-7 cells to GSK2606414, and it is likely to be due to the fact that these cells are already expressing high levels of FOXO3 and PERK (Supplementary Fig. S6).

### FOXO1/3/4-deletion downregulates PERK expression and enhances sensitivity to PERK inhibition

**Fig. 8:**
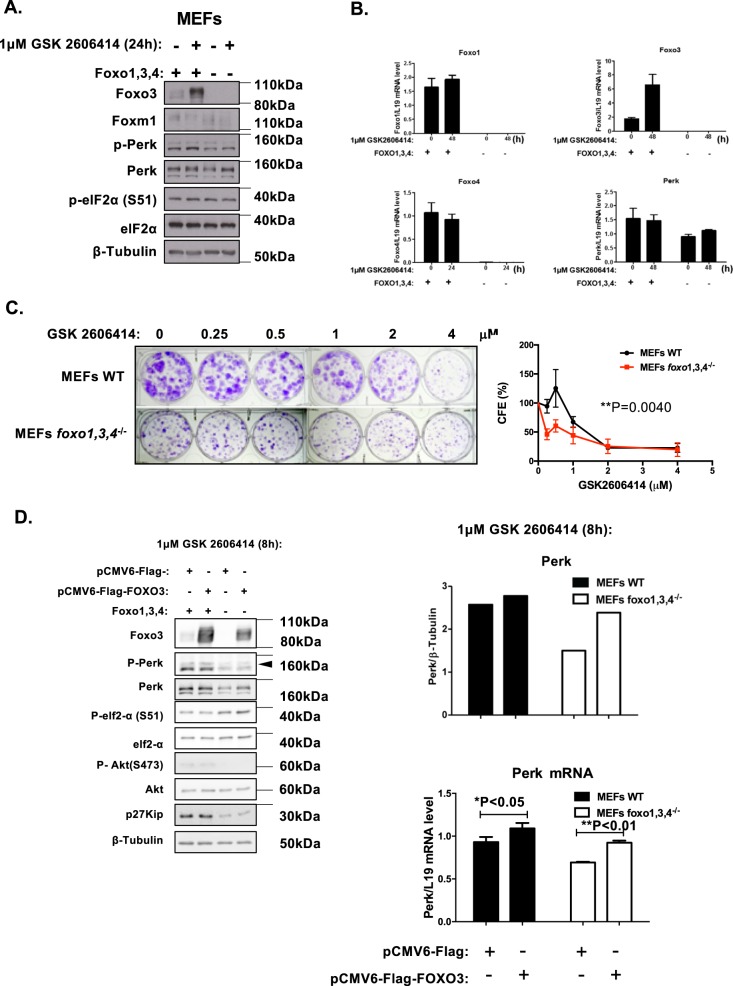
Effects of FOXO1,3,4 knock-out on protein and mRNA expression and GSK2606414 sensitivity. **a** Expression levels of protein and mRNA in WT and *Foxo*^*1/3/4*−/−^ MEFs in response to GSK2606414. Expression for P-Foxo3 (T32) (95 kDa), Foxo3 (85 kDa), P-Foxo1 (T24) (79 kDa), Foxo1 (79 kDa), Foxo4 (54 kDa), P-Perk (T981) (140 kDa) Perk (140 kDa), P-eIF2a (S51) (38 kDa), eIF2a (38 kDa) and β-Tubulin (55 kDa). **b** Foxo3, Foxo1, Foxo4 and Perk mRNA expression as determined by RT-qPCR. (two-tailed *t*-test). **c** MEFs WT and *Foxo*^*1/3/4*−/−^ treated with with 0, 0.25, 0.5, 1, 2 and 4 µM of PERK inhibitor GSK2606414 every 48 h for 15 days were then stained with crystal violet in clonogenic assays. Representative images of at least three independent experiments are shown. Data were displayed as means ± SEM (two-tailed *t*-test; Significant: *P < 0.05, **P < 0.01, ***P < 0.001). **d** Expression levels of PERK protein and mRNA were analysed by western blotting and RT-qPCR in WT and *Foxo*^*1/3/4*−/−^ MEFs after transfection with FOXO3 expression vector in the presence of 1 µM GSK2606414. Western blotting was performed to determine the protein expression levels for Foxo3 (85 kDa), P-Perk (T981) (140 kDa), Perk (140 kDa), P-eIF2a (S51) (38 kDa), eIF2a (38 kDa), P-Akt (S473) (60 kDa), Akt (60 kDa), p27Kip1 (27 kDa) and β-Tubulin (55 kDa) (left panel). The relative Perk expression levels were calculated by the ratio of Perk to tubulin expression (Top right panel). Perk mRNA levels were investigated by RT-qPCR, and the data were normalised with L19 RNA level and displayed as means ± SEM (*n* = 3; two-tailed *t*-test). Significant *P < 0.05, **P < 0.01. Representative RNA expression profiles of at least three independent experiments are shown (lower right panel)

To further validate our findings, we next examined the sensitivity of WT and *Foxo*^*1/3/4*−/−^ MEFs to the PERK inhibitor GSK2606414. Western blot and RT-qPCR analysis further confirmed FOXO1/3/4-deletion and that PERK expression is lower in *Foxo*^*1/3/4*−/−^ MEFs compared with WT MEFs (Fig. 8a, b). Clonogenic assay revealed that the *Foxo*^*1/3/4*−/−^ MEFs expressing lower levels of PERK are more sensitive to GSK2606414, further confirming the role of FOXO proteins in the modulating PERK expression and thereby GSK2606414 sensitivity. To confirm further that FOXO3 regulates PERK expression, we ectopic-expressed FOXO3 in FOXO1/3/4-deficient MEFs. Ectopic expression of FOXO3 in *Foxo*^*1/3/4*−/−^ MEFs induced high levels of cell death and only moderate levels of PERK induction (Supplementary Fig. S7). This is likely to be due to the negative feedback induction of PERK to phosphorylate and inactivate FOXO3. To circumvent the complications caused by the negative feedback loop, we ectopic-expressed FOXO3 in the presence of PERK inhibition to uncouple the negative feedback mechanism (Fig. 8d). The subsequent result revealed that reintroduction of FOXO3 substantially activates PERK transcription, especially in the presence of PERK inhibitor, thus confirming definitively that FOXO3 regulates PERK expression.

## Discussion

Anthracyclines and taxanes forms the backbone of most neo/adjuvant cancer treatments [29, 30]; however, the development of resistance to these cytotoxic agents has been a major obstacle to effective cancer therapy. ER stress can be induced by nutrient deprivation, hypoxia, and cytotoxic chemotherapy. It is also heightened in cancer cells due to a combination of factors, including deregulated gene transcription/translation, expression of truncated or altered proteins encoded by mutated genes and accumulation of damaged proteins caused by oxidative stress and chemotherapy [31]. To cope with ER stress, cancer cells activate the unfolded protein response (UPR) as a compensatory pro-survival mechanism [32]. In addition, chemoresistance has also been demonstrated to be strongly associated with the UPR [33, 34]. In concordance, the key ER stress sensor and UPR mediator, PERK has also been found to promote resistance to chemotherapy in cancer cells [18, 35–37], but the underlying mechanisms are not well understood. For example, colon cancer-resistant cancer cells showed a high-expression profile of P-PERK [18].

FOXO3 has been shown to mediate the cytotoxic effects of a variety of cytotoxic chemotherapeutic drugs, including epirubicin and paclitaxel [6, 38–42]. Previous studies have also demonstrated that FOXO3 expression is downregulated and its antagonist FOXM1 upregulated in most chemotherapy resistant cells [11, 43–48]. Consistent with this, in here we find FOXO3 expression and activity to be downregulated in epirubicin and paclitaxel-resistant MCF-7 breast cancer cells. Using ChIP, siRNA knockdown, and overexpression assays as well as *Foxo*^*1/3/4*−/−^ MEFs, we establish the endoplasmic reticulum (ER)-stress defence modulator PERK (eIF2AK3) as a direct downstream transcriptional target of FOXO3 through its promoter. Epirubicin can impact on the phosphorylation status of FOXO3 (T32) through a number of mechanisms. First, epirubicin can activate p38 MAPK and JNK [49], which can trigger FOXO3 phosphorylation (S7), nuclear relocalization, and FOXO3 (T32) dephosphorylation [21]. Moreover, epirubicin can also induce JNK, which can in turn repress AKT activity, leading to FOXO3 (T32) dephosphorylation [23]. In here, our data also show that epirubicin can also repress PERK activity to cause a downregulation in AKT activity and FOXO3 (T32) dephosphorylation. Conversely, overexpression of FOXO3 can induce the expression of PERK, which is subsequently phosphorylated and activated by endogenous cellular signalling. This notion is supported by the FOXO3 overexpression experiments showing that FOXO3 expression induces PERK expression accompanied by its phosphorylation/activation (Fig. 2b). The activated PERK can inactivate FOXO3 through AKT via a negative feedback loop.

In support of our finding, we also uncover that there is also a strong and significant correlation between FOXO3 and PERK mRNA levels in the Cancer Genome Atlas (TCGA) breast cancer patient datasets. This is likely to be due to the fact that FOXO3 regulates not only PERK expression but also its own transcription in a positive feed forward mechanism [14]. In fact, the strong correlation between FOXO3 and PERK expression at the mRNA level basically supports the notion that FOXO3 regulates PERK expression at the transcriptional level, which is by large independent of the breast cancer subtypes. Furthermore, a significant correlation between cytoplasmic FOXO3, PERK and P-eIF2α is also observed in a cohort of HER2+ breast cancer patient tissue samples. Notably, we discover that cytoplasmic and not nuclear FOXO3 correlates positively with PERK expression. Although nuclear staining is usually associated with activated FOXO3, our previous findings have shown that cytoplasmic rather than nuclear FOXO3 straining represents functional FOXO3 and is a better marker for its activity [22]. In agreement, constitutively nuclear FOXO3 localisation indicates deregulated FOXO3 function and also predicts poor survival in breast cancer samples [22]. Other studies also show that cytoplasmic FOXO3 is also a favourable independent prognostic factor in breast cancer [27]. eIF2α phosphorylation (P-eIF2α) is directly mediated by PERK and therefore reflects PERK activity. This cytoplasmic FOXO3 and PERK staining also significantly corresponds with P-eIF2α staining. In agreement with the notion that cytoplasmic FOXO3 being the functional form of the tumour suppressor, previous findings also have demonstrated that PERK and P-eIF2α levels are significantly associated with higher histological grades, higher numbers of tumour-infiltrating lymphocytes (TILs), which are good prognostic markers in human epidermal growth factor receptor 2 (HER2)-positive breast cancer tissues. We have previously shown that HER2 expression can promote FOXO3 cytoplasmic localisation via activating AKT [11, 50, 51], and this could account for the observation that 95.7% of the 447 HER2+ breast cancer cases have cytoplasmic FOXO3 expression [52]. The high cytoplasmic FOXO3 expression rates in this panel of HER2+ breast cancer samples could also be the reason why a good correlation analysis between PERK and cytoplasmic FOXO3 expression can be made. Univariate and multivariate analyses indicate that both cytoplasmic FOXO3 and PERK are not independent prognostic factors (Supplementary Fig. S8). This could be due to the fact that the anti-PERK drug has not been a mainstay of breast cancer treatment, in particular this panel of cancer samples.

The high levels of the activated P-PERK in the drug-resistant breast cancer cell lines point to elevated UPR in response to heightened ER-stress in the drug-resistant cells. This is likely to be caused by the increased accumulation of misfolded/unfolded gene products as a result of higher metabolic rates and mutation frequencies in these drug-resistant cancer cells. This adaptive dependence on higher PERK activity to combat elevated ER-stress coupled to the intrinsically low PERK levels as a result of low expression and activity of its regulator FOXO3 in these drug-resistant breast cancer cells renders them particularly vulnerable to PERK inhibition. This notion is supported by our PERK inhibitor titration assays and time course experiments showing that PERK activity, as revealed by P-PERK (T981), is more susceptible to inhibition by increasing concentrations of GSK2606414 in the drug-resistant cells compared to MCF-7 cells because of their low PERK levels. The latter is further supported by our findings that *Foxo*^*1/3/4*−/−^ MEFs, which express lower PERK levels but more resistant to other conventional chemotherapeutic drugs are more sensitivity to PERK inhibition compared with their wild-type counterparts. In addition, our experiments also show that although overexpression of FOXO3 decreases the overall clonogenicity of the paclitaxel- and epirubicin-resistant cells, it increases their resistance to the PERK inhibitor.

PERK has previously been shown to be able to phosphorylate FOXO3 directly at S261, S298, S301, S303 and S311 to promote FOXO3 nuclear relocation and activation [25]. In contrast, our results show that pharmacological inhibition of PERK by GSK2606414 causes FOXO3 dephosphorylation at (T32), the AKT phosphorylation site, suggesting that epirubicin and paclitaxel treatment mediates FOXO3 dephosphorylation (T32) and activation, at least in part, through inhibiting PERK activity. Consistent with our observation, PERK has previously been shown to possess lipid kinase activity that promotes mTOR activity and AKT phosphorylation (S473)/activation [53]. Moreover, pharmacological inhibition of PERK has also been reported to result in its sequestration of the PTEN-containing Vault complex in the cytoplasm, leading to increased PTEN and therefore repressed PI3K-AKT activity [15]. In consequence, our results also suggest that constitutive PERK activation can negatively regulate FOXO3 activity indirectly via AKT to promote cancer survival and drug resistance. Notably, western blot analysis showed that treatment with the specific PERK inhibitor GSK2606414 did not repress PDK1 phosphorylation/activity in all three MCF-7 cell lines (Fig. 5), indicating that GSK2606414 is unlikely to dephosphorylate and activate FOXO3 through repressing PDK1. This is consistent with the observation that GSK2606414 does not repress AKT phosphorylation (T308), which is the phosphorylation target of PDK1 and a reliable readout of PDK1 activity [54]. In addition, in the original characterisation of the inhibitor, the authors performed kinase selectivity profiling of 294 cellular kinases and showed that GSK2606414 is a specific PERK inhibitor [55]. Particularly, GSK2606414 does not inhibit AKT and PDK activity at all even at the 10 μM level. Thus, PERK can potentially mediate FOXO3 phosphorylation and inhibition via mTORC2 (mechanistic target of rapamycin complex 2) and/or the related DNA-dependent protein kinase (DNA-PK), which have been shown to be responsible for AKT (S473) phosphorylation [54]. Conversely, chemotherapeutic drugs and PERK inhibitors can activate FOXO3 via inhibiting PERK-AKT axis in breast cancer cells. This may suggest that the higher PERK activity observed in the drug-resistant cells contributes to further FOXO3 repression and thereby lower PERK expression. Therefore, in addition, our data also suggest that the regulation of FOXO3 by PERK is predominantly mediated through the phosphorylation (T32) and inactivation by AKT in the drug-sensitive cancer cells. The present study also advocates the PERK-AKT-FOXO3 axis may contribute to the cytotoxic action of paclitaxel and epirubicin in the cancer cells.

In conclusion, our study establishes PERK (eIF2AK3) as a direct downstream transcriptional target of FOXO3. Our work also identifies the adaptive response of low FOXO3 expression to boost drug survival and the overdependence on elevated PERK activity to overcome heightened ER-stress, as an acquired vulnerability of the drug-resistant cancer cells. The fact that there is a strong and significant correlation between PERK and FOXO3 expression in the breast cancer patient samples suggest that this FOXO3-PERK regulatory axis is preserved in most breast cancers. Our present work provides the explanation for the reason why drug-resistant cancer cells are prone to PERK inhibition and further suggests that PERK inhibitors provide a therapeutic opportunity for targeting cancer, in particular the drug-resistant cancer cells.

## Materials and methods

### Patients and tissue specimens

The patient tissue samples came from a study consisting of 447 HER2-positive breast cancer patients who underwent surgery for primary breast cancer between 2006 and 2011, at the Department of Pathology, Asan Medical Centre, Seoul, Korea. Formalin-fixed, paraffin-embedded tissue samples from these preoperatively chemo- and radiotherapy naive patients were available for analysis as previously described [56]. See also Supplementary Materials and Methods.

### Cell lines and culture conditions

MCF-7, a human breast cancer cell line, originated in the American Type Culture Collection (Manassas, VA, USA) and was acquired from the Cell Culture Service, Cancer Research UK (London, UK), where it was tested and authenticated. The MCF-7-Epi^R^ (resistant to epirubicin) [57] and MCF-7-Tax^R^ (resistant to paclitaxel) [58] cell lines were generated in the lab (Imperial College London, Hammersmith hospital, UK) by culturing parental MCF-7 cells in gradually increased drug concentrations until they obtained resistance to 100 μmol/L of the drugs [45]. Mouse embryonic fibroblasts (MEFs) WT and *Foxo*^*1/3/4*−/−^ were kind gifts from Professor Boudewijn Burgering, UMC, Utrecht, the Netherlands and have previously been described [13, 59, 60]. All cell lines were cultured in Dulbecco’s Modified Eagle’s Medium (Sigma-Aldrich, Irvine, UK) supplemented with 10% fetal calf serum (First Link Ltd, Birmingham, UK), 4 mM glutamine and 100 U/mL penicillin/streptomycin (Sigma-Aldrich). All cell lines were incubated at 37 °C with 10% CO_2_. The drug resistance of MCF-7-Tax^R^ cells was maintained with 0.05 μM Paclitaxel (Hospira, Maidenhead, UK), and the MCF-7-Epi^R^ cells in 17 μM epirubicin (Accord, Middlesex, UK).

### Transfection

Plasmid transfections were performed with FuGENE® HD (Promega) according to the manufacturer’s protocol. For siRNA, cells were transfected with ON-TARGETplus SMARTpool siRNAs (GE Dharmacon, Horizon Discovery LTD, UK) using Oligofectamine (Invitrogen, Thermo Fisher Scientific, UK) following the manufacturer’s instructions. After 48 h of transfection, cells were treated with epirubicin, GSK2606414 or paclitaxel for the time points indicated and collected for analysis by western blot, RT-qPCR, SRB or clonogenic assays.

### Western blotting

Western blot was performed on whole-cell extracts as previously described [61] using the antibodies mentioned. FOXM1 (c-20; sc-502), P-PERK (Thr 981) (sc-32577), p27^Kip1^ (sc-528) and β-tubulin H-235; sc-9104) antibodies were purchased from Santa Cruz Biotechnology (CA, USA). The P-FOXO1 (Thr24)/FOXO3 (Thr32) (CST# 9464) and FOXO3 (CST#2497), PERK (CST#3192), FOXO1 (CST#9454), FOXO4 (CST #9472), Bim (CST#2933), P-AKT (S473) (CST#9271), P-AKT(T308) (CST#9275), AKT (CST#9272) P-PDK-1 (S241) (CST#3061), PDK-1 (CST#3062) and eIF2α (CST#5324) were purchased from Cell Signaling Technology (New England Biolabs Ltd. Hitchin, UK). The P-elF2α antibody (ab32157; Abcam; Cambridge, UK). The primary antibodies (1:1000) were detected using horseradish peroxidase-conjugated secondary antibody (1:2000, DAKO, Glostrup, Denmark) and visualised using the ECL detection system (PerkinElmer Ltd, Beaconsfield, UK).

### Quantitative real-time PCR (RT-qPCR)

RT-qPCR analysis was performed as described [61]. Total RNA was extracted using the RNeasy Mini kit (Qiagen, Hilden, Germany). Complementary DNA was reverse-transcribed into cDNA using SuperScript Transcriptase III (Invitrogen) according to the manufacturer’s protocol. Gene expressions were quantified via RT-qPCR, using Power SYBR Green PCR Master Mix (Applied Biosystems, Fisher Scientific UK Ltd, Loughborough, UK) and a standard curve as previously described [62]. See also Supplementary Materials and Methods.

### Chromatin immunoprecipitation (ChIP)

ChIP analysis was performed as described [61]. The cell lines were transfected with pCMV5-FOXO3 and the empty vector pCMV5 for 24 h, after which FOXO3-overexpressing cells were collected for the ChIP assay, as previously described. For the immunoprecipitation, 4 µg of either IgG (P0447, DAKO) and FOXO3 (ab12162; Abcam) antibodies were added to the precleared samples. The control region used was the promoter region of PERK where there are no known FOXO-binding motifs and the ChIP signal for FOXO3 is very low compared with ChIP peaks from the ChIP-Seq data. See also Supplementary Materials and Methods.

### Sulforhodamine B (SRB) assays

The sulforhodamine B assay was used for analysing short-term cell viability in drug-treated cells following GSK2606414 treatment and/or FOXO3 overexpression and has previously been described [61]. See also Supplementary Materials and Methods.

### Clonogenic assay

Total of 3000 MCF-7, MCF-7-Epi^R^, MCF-7-Tax^R^ cells were seeded into six-well plates and left overnight for adherence, after which they were treated with increasing concentrations of GSK2606414 and has previously been described [61]. See also Supplementary Materials and Methods.

### Statistical analysis

The correlations between protein expression in patient samples were analysed by Chi-square and bi-variate Pearson Correlation statistical analysis using SPSS 16.0 (Imperial College London, Software Shop, UK). Other results shown are representative of three independent experiments, which were each performed in triplicate (presented as the mean ± SEM). GraphPad Prism was used for statistical analysis (version 5, San Diego, CA, USA), and two-tailed Student’s *t*-test was used to compare the means. For comparisons between groups of more than two unpaired values, one-way analysis of variance (ANOVA) was used. Two-way ANOVA was used between groups of two variables, and was considered significant when *P < 0.05 s, **P < 0.01 and ***P < 0.001. ns for non-significant.

## Supplementary information

Supplementary Materials and Methods

Supplementary Figure S1

Supplementary Figure S2

Supplementary Figure S3

Supplementary Figure S4

Supplementary Figure S5

Supplementary Figure S6

Supplementary Figure S7

Supplementary Figure S8

Supplementary Figure Legends
