# Supplementary material for: Regulation of PERK expression by FOXO3: a vulnerability of drug-resistant cancer cells
Source: Oncogene. 2019 Jul 16;38(36):6382–98. doi: 10.1038/s41388-019-0890-7 (PMC6756075; doi:10.1038/s41388-019-0890-7)
Supplement: Supplementary file 1 — Supplementary Materials and Methods [file 41388_2019_890_MOESM1_ESM.docx]

**Supplementary Materials and Methods**

**Patients and tissue specimens**

The patient tissue samples came from a study consisting of 447 HER2-positive breast cancer patients who underwent surgery for primary breast cancer between 2006 and 2011, at the Department of Pathology, Asan Medical Center, Seoul, Korea. Formalin-fixed, paraffin-embedded tissue samples from these preoperatively chemo- and radiotherapy naive patients were available for analysis as previously described ^47^. The expression levels of standard biomarkers, including estrogen receptor (ER), progesterone receptor (PR), and HER2, were reviewed by immunohistochemical staining at the time of diagnosis, independently by two pathologists. HER2-overexpressing tumours were defined as those that score of 3+ by immunohistochemistry (IHC) or by gene amplification using either fluorescence *in situ* hybridization or silver *in situ* hybridization ^48^. Exemption from informed consent after the de-identification of information was approved by the Institutional Review Board. Tissue microarray sections were evaluated with an automated immunohistochemical staining device (Benchmark XT; Ventana Medical Systems, Tucson, AZ, USA). Antibodies against FOXO3 (1:400 dilution; EMD Millipore, Billerica, MA, USA), PERK (1:200; #5683; Cell Signaling Technology, Danvers, MA, USA), p-eIF2α (1:1000; ab32157; Abcam, Cambridge, UK) were used. Each case was evaluated by estimating the percentage and staining intensity (negative, 0; weak, 1; moderate, 2; and strong, 3) of tumour cells showing a cytoplasmic or nuclear FOXO3, PERK and p-eIF2α. We then classified the expression levels as high or low based on the mean staining intensity value obtained by the multiplication of each protein. Statistical analyses were carried out using SPSS ver. 18 (SPSS Inc., Chicago, IL) and R statistical software. The chi-squared test, Spearman’s and correlation were employed, as appropriate. All tests were two-sided and were done at significance level of α=0.05.

**Quantitative real time PCR (RT-qPCR)**

**RT-qPCR** analysis was performed as described ^53^. Total RNA was extracted using the RNeasy Mini kit (Qiagen, Hilden, Germany). Complementary DNA was reverse-transcribed into cDNA using SuperScript Transcriptase III (Invitrogen) according to the manufacturer’s protocol. Gene expressions were quantified via RT-qPCR, using Power SYBR Green PCR Master Mix (Applied Biosystems, Fisher Scientific UK Ltd, Loughborough, UK) and a standard curve as previously described ^54^. L19, a housekeeping gene, was used as an internal control for normalization. Human primer sequences are L19-F 5’ GCGGAAGGGTACAGCCAAT3’, L19-R 5’ GCAGCCGGCGCAAA 3’, FOXO3-F 5’ TCTACGAGTGGATGGTGCGTT 3’, FOXO3-R 5’ CGACTATGCAGTGACAGGT3’, FOXM1-F 5’ TGCAGCTAGGGATGTGAATCTTC 3’ , FOXM1-R 5’ GGAGCCCAGTCCATCAGAACT 3’, PERK-F 5’ TGGCCACTTTGAACTTCGGTA 3’, PERK-R 5’ CCACCCGGTTTAAAGGTGCT 3’, FOXO1-F 5’ TGGACATGCTCAGCAGACATC 3’, FOXO1-R 5’ TTGGGTCAGGCGGTTCA 3’, p27^Kip1^-F 5’CATTTGGTGGACCCAAAGAC3’,p27^Kip1^-R 5’CTTCTGAGGCCAGGCTTCTT3’. For mouse primer sequences are L19-F 5’GGTGCTTCCGATTCCAGAGT3’, L19-R 5’CCCATTCCCTGATCGCTTGA 3’, Foxo3-F 5’ CCGGACAAACGGCTCACT 3’, Foxo3-R 5’ GGCACACAGCGCACCAT 3’, Foxo4-F 5’AGGACAAGGGTGACAGCAAC 3’, Foxo4-R 5’GGTTCAGCATCCACCAAGAG 3’, Perk-F 5’GGATGTCGCCGATGGGATAG 3’, Perk-R 5’CGAAGTTCAAAGTGGCCAACA 3’, Foxo1-F 5’AAGAGCGTGCCCTACTT-CAA 3’, Foxo1-R 5’TCCTTCA-TTCTGCACTCGAA 3’.

**Chromatin immunoprecipitation (ChIP)**

ChIP analysis was performed as described ^53^. The cell lines were transfected with pCMV5-FOXO3 and the empty vector pCMV5 for 24 h , after which FOXO3-overexpressing cells were collected for the ChIP assay, as previously described. For the immunoprecipitation, 4 µg of either IgG (P0447, DAKO) and FOXO3 (ab12162; Abcam) antibodies were added to the precleared samples. Then, a PCR purification kit (Qiagen) was used to purify the DNA according to the manufacturer’s instructions. For PCR reaction, 2.5 µL DNA from each sample, 0.5 µL of mix of primers (50 nM final concentration), 5 µL SYBR green master mix (Applied Biosystems) and 2 µL DEPC-treated water per well were used. The reaction was run in 7900 HT Fast Real-time PCR System (Applied Biosystems) and the cycling program was 95 °C for 10 min followed by 40 cycles of 95 °C for 15 s, 60 °C for 30 s and 95 °C for 30 s, followed by a dissociation step. The pair of primers used for ChIP was: PERK-F 5′ GATGGCAGTGACCTGTGACA 3′ and PERK-R 5′ AGTCTTCTCCACTCTGCCCT3′. The control primers are Actin control-F 5′ AGCGCGGCTACAGCTTCA3′ and Actin control-R 5′ CGTAGCACAGCTTCTCCTTAATGT 3’. All experiments were done in triplicates and results were normalized to the IgG antibody.

**Sulforhodamine B (SRB) assays**

The sulforhodamine B assay was used for analysing short-term cell viability in drug-treated cells following GSK2606414 treatment and/or FOXO3 overexpression and has previously been described ^53^. Briefly, 3000 cells were seeded in 96-well plates and left to adhere for 24 h, after which specific doses of GSK2606414 were added. Cells were maintained in culture for additional 24, 48 and 72 h, fixed with 100 µl of 40% trichloroacetic acid for 1 h at 4 °C, washed 5 times with distilled water and stained with 100 µl SRB solution (0.4% SRB diluted in 1% acetic acid) for 1 h. Afterwards, plates were washed 5 times with 1% acetic acid and air-dried. Protein-bound dye was solubilized in 100 µl of 10 mM Tris solution and optical density was measured in a microplate reader at 492 nm (Sunrise, Tecan, Dorset, UK).

**Clonogenic assay**

Total of 3000 MCF-7, MCF-7-Epi^R^ , MCF-7-Tax^R^ cells were seeded into six-well plates and left overnight for adherence, after which they were treated with increasing concentrations of GSK2606414 and has previously been described ^53^. Briefly after 48 h of incubation with the drug, cells were cultured in fresh drug-free media and grown for around 14 days until colony formation. Colonies were washed 3 times with PBS and fixed with 4% formaldehyde for 15 min at room temperature. After 3 additional washes with PBS, colonies were stained with 0.5% crystal violet (Sigma Aldrich) for 1 h, washed with flowing water, air-dried and quantified using ImageJ (<https://imagej.nih.gov>).
