# Supplementary material for: Regulation of PERK expression by FOXO3: a vulnerability of drug-resistant cancer cells
Source: Oncogene. 2019 Jul 16;38(36):6382–98. doi: 10.1038/s41388-019-0890-7 (PMC6756075; doi:10.1038/s41388-019-0890-7)
Supplement: Supplementary file 2 — Supplementary Figure S1 [file 41388_2019_890_MOESM2_ESM.pptx]

## Slide 1
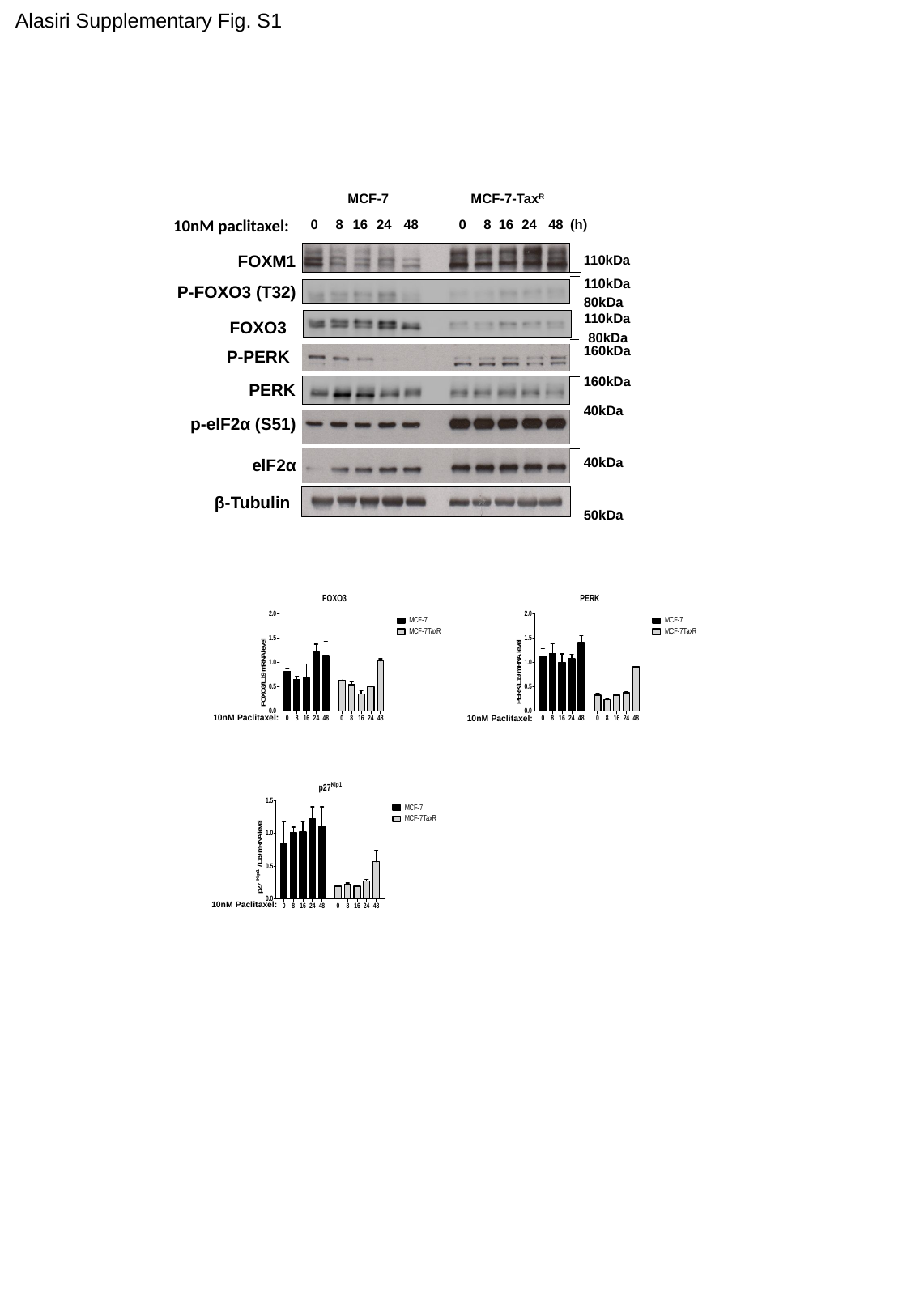

Alasiri Supplementary Fig. S1
MCF-7
MCF-7-TaxR
10nM paclitaxel:
0
8
16
24
48
0
8
16
24
48
(h)
FOXM1
110kDa
110kDa
P-FOXO3 (T32)
80kDa
110kDa
FOXO3
80kDa
160kDa
P-PERK
160kDa
PERK
40kDa
 p-elF2α (S51)
40kDa
elF2α
β-Tubulin
50kDa
10nM Paclitaxel:
10nM Paclitaxel:
10nM Paclitaxel:
