# Supplementary material for: Regulation of PERK expression by FOXO3: a vulnerability of drug-resistant cancer cells
Source: Oncogene. 2019 Jul 16;38(36):6382–98. doi: 10.1038/s41388-019-0890-7 (PMC6756075; doi:10.1038/s41388-019-0890-7)
Supplement: Supplementary file 3 — Supplementary Figure S2 [file 41388_2019_890_MOESM3_ESM.pptx]

## Slide 1
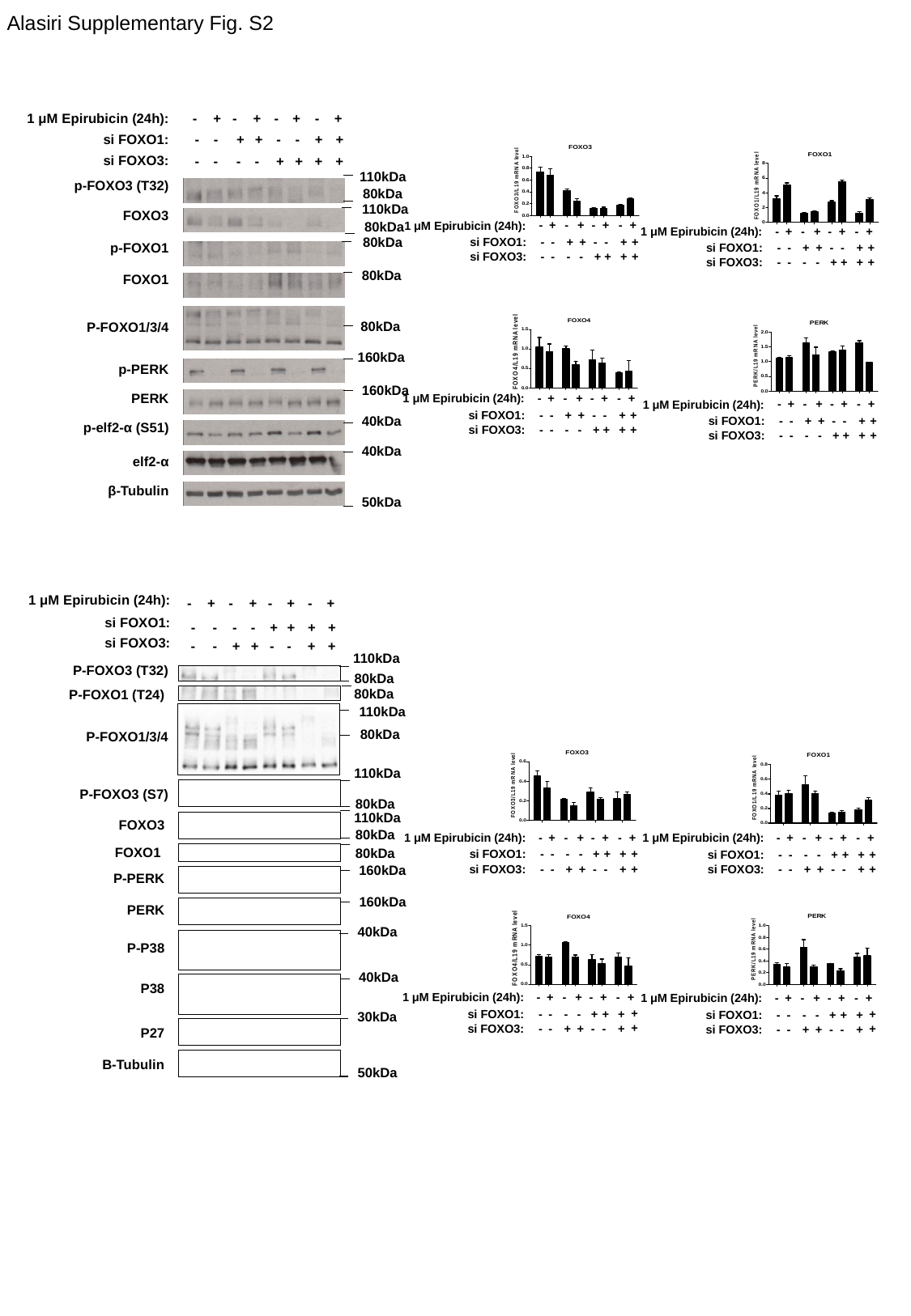

Alasiri Supplementary Fig. S2
-
+
-
+
-
+
-
+
1 μM Epirubicin (24h):
si FOXO1:
-
-
+
+
-
-
+
+
-
+
-
+
-
+
-
+
1 μM Epirubicin (24h):
si FOXO1:
-
-
+
+
-
-
+
+
si FOXO3:
-
-
-
-
+
+
+
+
si FOXO3:
-
+
-
+
-
+
-
+
1 μM Epirubicin (24h):
si FOXO1:
-
-
+
+
-
-
+
+
si FOXO3:
-
-
-
-
+
+
+
+
-
-
-
-
+
+
+
+
110kDa
p-FOXO3 (T32)
80kDa
110kDa
FOXO3
80kDa
80kDa
p-FOXO1
80kDa
FOXO1
80kDa
P-FOXO1/3/4
-
+
-
+
-
+
-
+
1 μM Epirubicin (24h):
si FOXO1:
-
-
+
+
-
-
+
+
si FOXO3:
-
-
-
-
+
+
+
+
-
+
-
+
-
+
-
+
1 μM Epirubicin (24h):
si FOXO1:
-
-
+
+
-
-
+
+
si FOXO3:
-
-
-
-
+
+
+
+
160kDa
p-PERK
160kDa
PERK
40kDa
 p-elf2-α (S51)
40kDa
elf2-α
β-Tubulin
50kDa
1 μM Epirubicin (24h):
-
+
-
+
-
+
-
+
si FOXO1:
-
-
-
-
+
+
+
+
si FOXO3:
-
-
+
+
-
-
+
+
110kDa
P-FOXO3 (T32)
80kDa
80kDa
P-FOXO1 (T24)
110kDa
80kDa
P-FOXO1/3/4
1 μM Epirubicin (24h):
-
+
-
+
-
+
-
+
si FOXO1:
-
-
-
-
+
+
+
+
si FOXO3:
-
-
+
+
-
-
+
+
1 μM Epirubicin (24h):
-
+
-
+
-
+
-
+
si FOXO1:
-
-
-
-
+
+
+
+
si FOXO3:
-
-
+
+
-
-
+
+
110kDa
P-FOXO3 (S7)
80kDa
110kDa
FOXO3
80kDa
FOXO1
80kDa
160kDa
P-PERK
160kDa
PERK
1 μM Epirubicin (24h):
-
+
-
+
-
+
-
+
+
si FOXO1:
-
-
-
-
+
+
+
+
si FOXO3:
-
-
+
+
-
-
+
1 μM Epirubicin (24h):
-
+
-
+
-
+
-
+
+
si FOXO1:
-
-
-
-
+
+
+
+
si FOXO3:
-
-
+
+
-
-
+
40kDa
P-P38
40kDa
P38
30kDa
P27
B-Tubulin
50kDa
