# Supplementary material for: Regulation of PERK expression by FOXO3: a vulnerability of drug-resistant cancer cells
Source: Oncogene. 2019 Jul 16;38(36):6382–98. doi: 10.1038/s41388-019-0890-7 (PMC6756075; doi:10.1038/s41388-019-0890-7)
Supplement: Supplementary file 4 — Supplementary Figure S3 [file 41388_2019_890_MOESM4_ESM.pptx]

## Slide 1
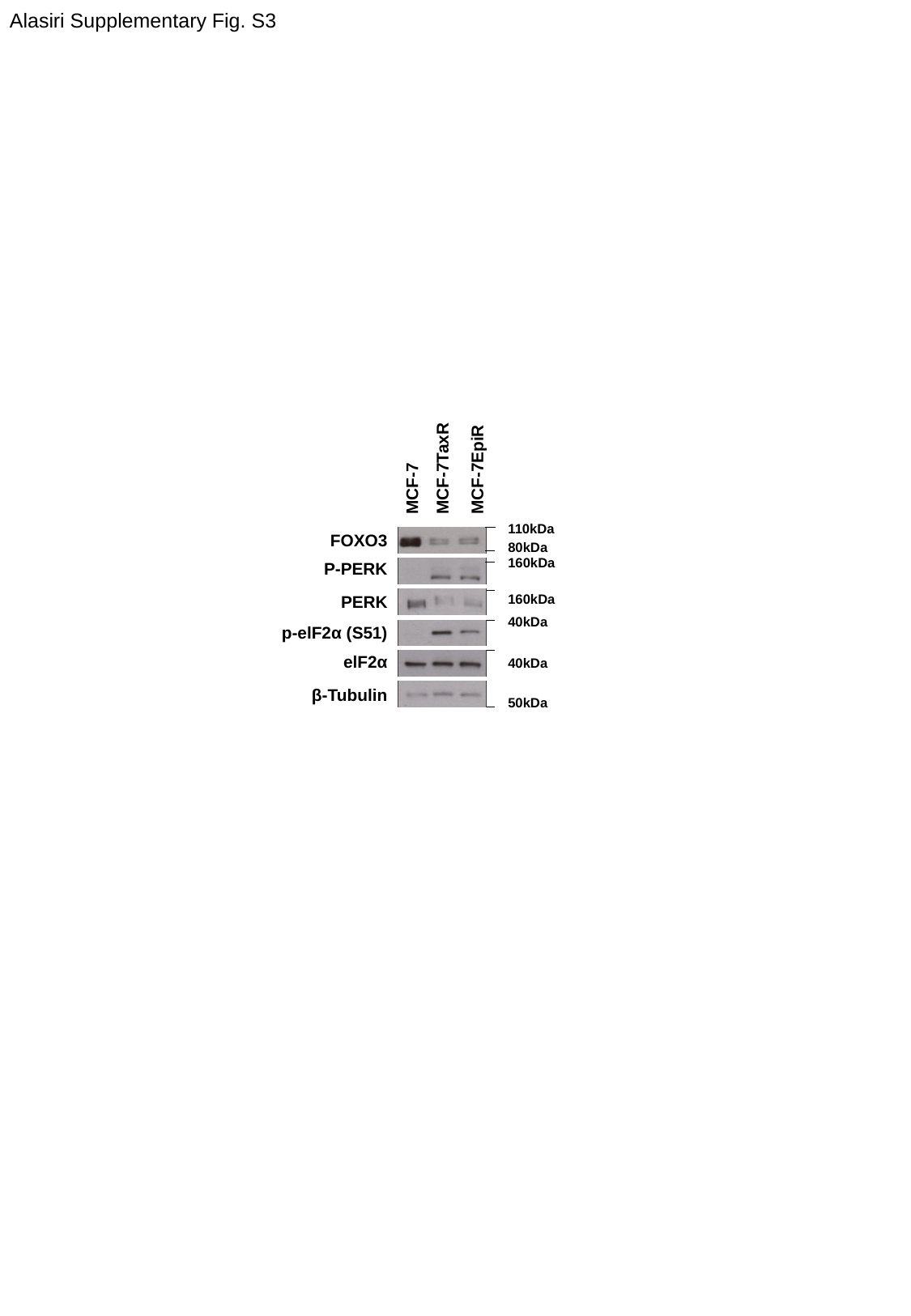

Alasiri Supplementary Fig. S3
MCF-7
MCF-7TaxR
MCF-7EpiR
110kDa
FOXO3
80kDa
160kDa
P-PERK
160kDa
PERK
40kDa
 p-elF2α (S51)
elF2α
40kDa
β-Tubulin
50kDa
