# Supplementary figures and images for: Regulation of PERK expression by FOXO3: a vulnerability of drug-resistant cancer cells
Source: Oncogene. 2019 Jul 16;38(36):6382–98. doi: 10.1038/s41388-019-0890-7 (PMC6756075; doi:10.1038/s41388-019-0890-7)

## Slide 1
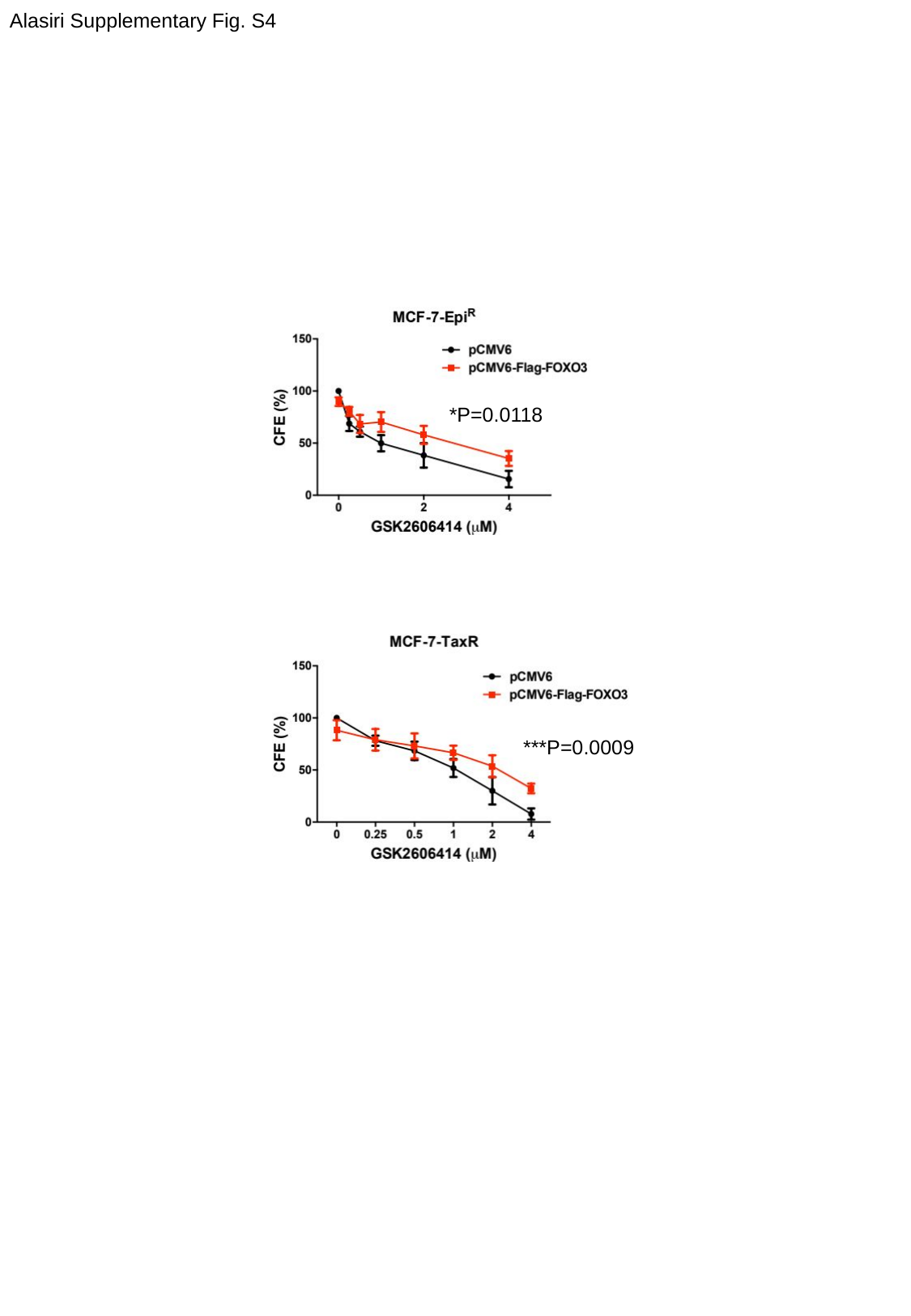

Alasiri Supplementary Fig. S4
*P=0.0118
***P=0.0009

Supplement: Supplementary file 5 — Supplementary Figure S4 [file 41388_2019_890_MOESM5_ESM.pptx]
