# Supplementary material for: Regulation of PERK expression by FOXO3: a vulnerability of drug-resistant cancer cells
Source: Oncogene. 2019 Jul 16;38(36):6382–98. doi: 10.1038/s41388-019-0890-7 (PMC6756075; doi:10.1038/s41388-019-0890-7)
Supplement: Supplementary file 6 — Supplementary Figure S5 [file 41388_2019_890_MOESM6_ESM.pptx]

## Slide 1
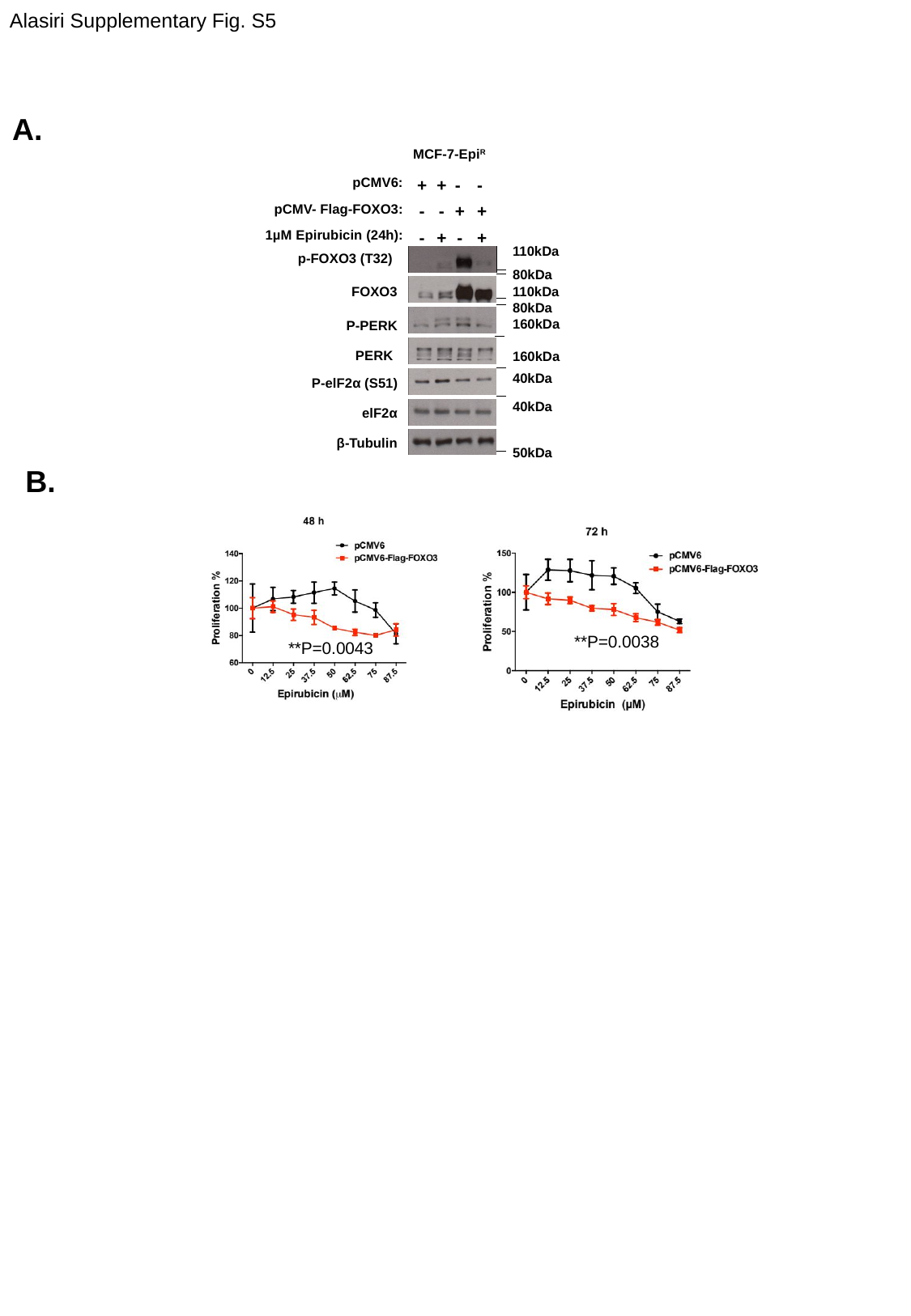

Alasiri Supplementary Fig. S5
A.
MCF-7-EpiR
pCMV6:
+
+
-
-
pCMV- Flag-FOXO3:
-
-
+
+
1µM Epirubicin (24h):
-
+
-
+
110kDa
p-FOXO3 (T32)
80kDa
FOXO3
110kDa
80kDa
160kDa
P-PERK
PERK
160kDa
40kDa
 P-elF2α (S51)
40kDa
elF2α
β-Tubulin
50kDa
B.
**P=0.0038
**P=0.0043
| |
| --- |
