# Supplementary material for: Regulation of PERK expression by FOXO3: a vulnerability of drug-resistant cancer cells
Source: Oncogene. 2019 Jul 16;38(36):6382–98. doi: 10.1038/s41388-019-0890-7 (PMC6756075; doi:10.1038/s41388-019-0890-7)
Supplement: Supplementary file 7 — Supplementary Figure S6 [file 41388_2019_890_MOESM7_ESM.pptx]

## Slide 1
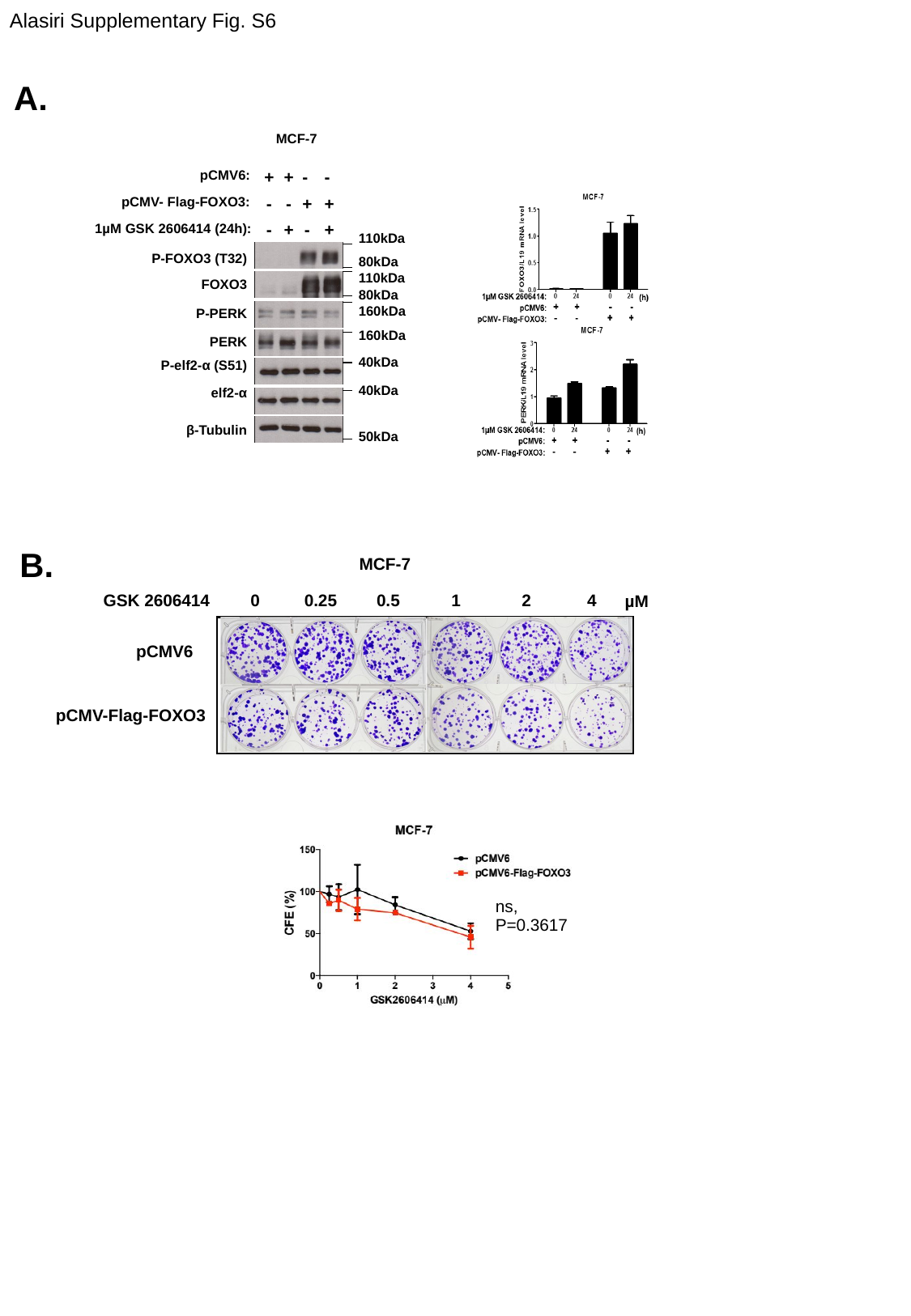

Alasiri Supplementary Fig. S6
A.
MCF-7
pCMV6:
+
+
-
-
pCMV- Flag-FOXO3:
-
-
+
+
-
+
-
+
1µM GSK 2606414 (24h):
110kDa
P-FOXO3 (T32)
80kDa
110kDa
FOXO3
80kDa
160kDa
P-PERK
160kDa
PERK
40kDa
 P-elf2-α (S51)
40kDa
elf2-α
β-Tubulin
50kDa
B.
MCF-7
GSK 2606414
0
0.25
0.5
1
2
4
µM
pCMV6
pCMV-Flag-FOXO3
| ns, P=0.3617 |
| --- |
