# Supplementary material for: Regulation of PERK expression by FOXO3: a vulnerability of drug-resistant cancer cells
Source: Oncogene. 2019 Jul 16;38(36):6382–98. doi: 10.1038/s41388-019-0890-7 (PMC6756075; doi:10.1038/s41388-019-0890-7)
Supplement: Supplementary file 8 — Supplementary Figure S7 [file 41388_2019_890_MOESM8_ESM.pptx]

## Slide 1
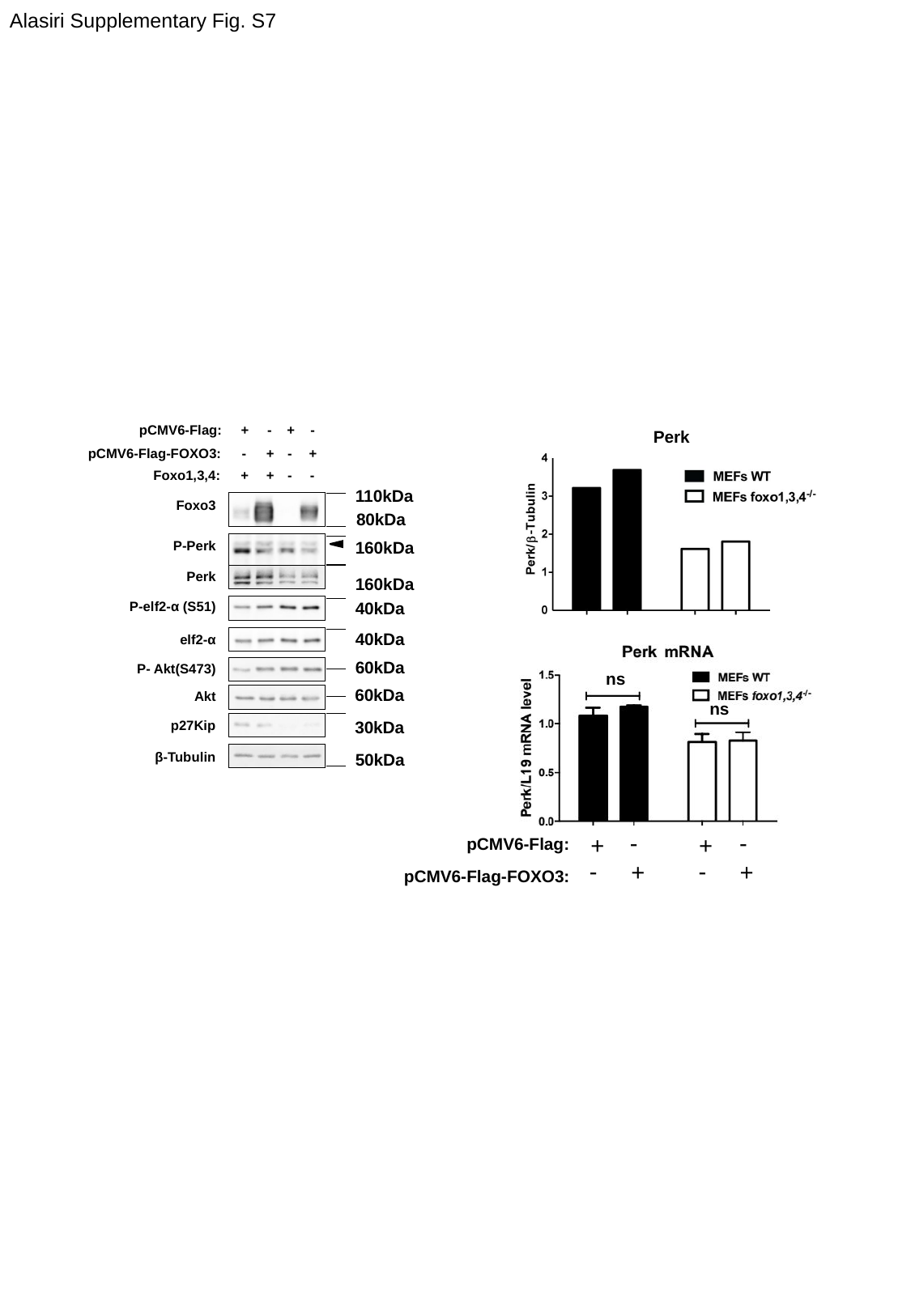

Alasiri Supplementary Fig. S7
pCMV6-Flag:
+
-
+
-
Perk
pCMV6-Flag-FOXO3:
-
+
-
+
Foxo1,3,4:
+
+
-
-
110kDa
Foxo3
80kDa
P-Perk
160kDa
Perk
160kDa
40kDa
 P-elf2-α (S51)
40kDa
elf2-α
60kDa
P- Akt(S473)
ns
60kDa
Akt
ns
30kDa
p27Kip
β-Tubulin
50kDa
-
-
+
+
pCMV6-Flag:
-
-
+
+
pCMV6-Flag-FOXO3:
