# Supplementary material for: Regulation of PERK expression by FOXO3: a vulnerability of drug-resistant cancer cells
Source: Oncogene. 2019 Jul 16;38(36):6382–98. doi: 10.1038/s41388-019-0890-7 (PMC6756075; doi:10.1038/s41388-019-0890-7)
Supplement: Supplementary file 9 — Supplementary Figure S8 [file 41388_2019_890_MOESM9_ESM.pptx]

## Slide 1
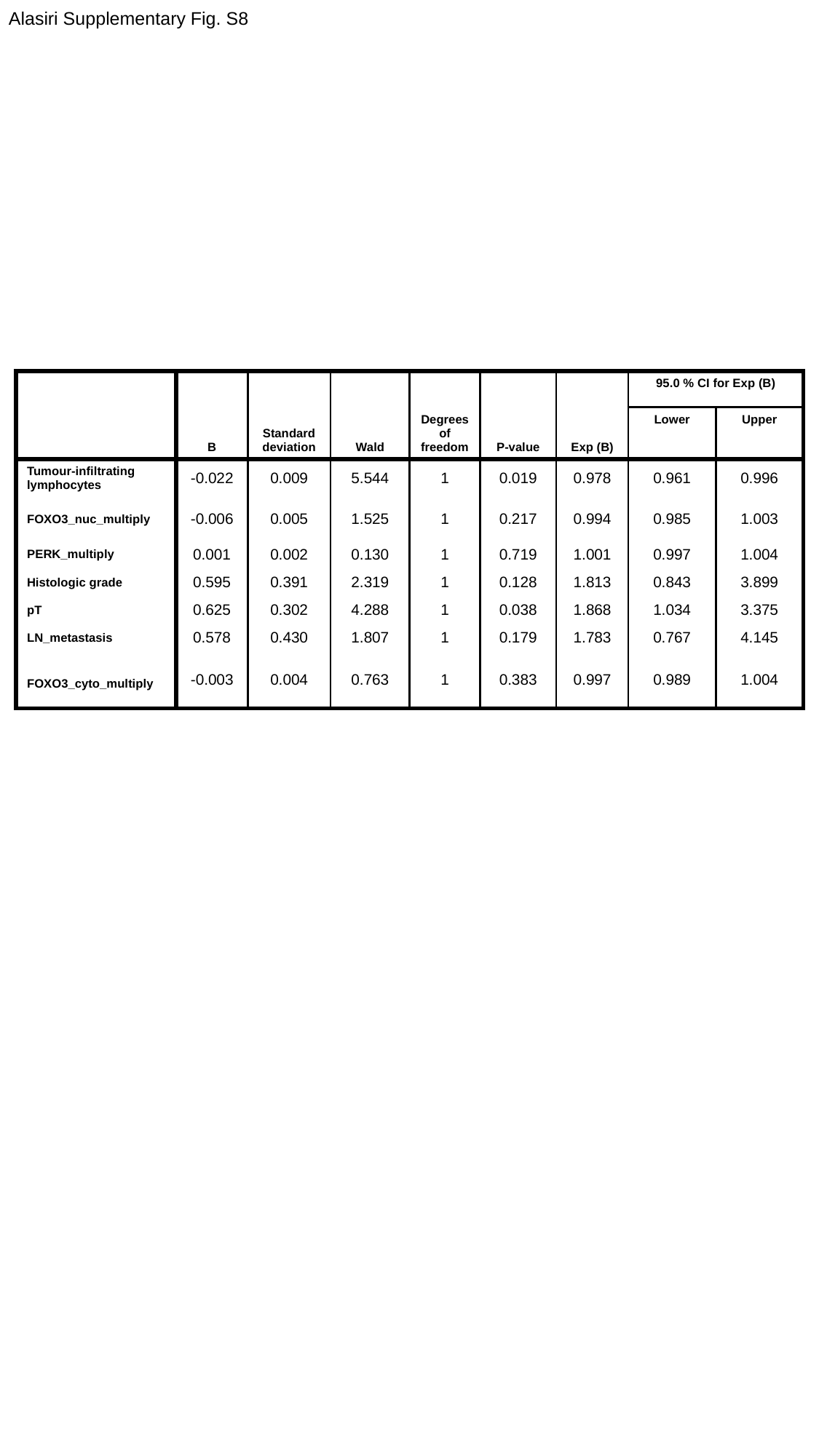

Alasiri Supplementary Fig. S8
| | B | Standard deviation | Wald | Degrees of freedom | P-value | Exp (B) | 95.0 % CI for Exp (B) | |
| --- | --- | --- | --- | --- | --- | --- | --- | --- |
| | | | | | | | Lower | Upper |
| Tumour-infiltrating lymphocytes | -0.022 | 0.009 | 5.544 | 1 | 0.019 | 0.978 | 0.961 | 0.996 |
| FOXO3\_nuc\_multiply | -0.006 | 0.005 | 1.525 | 1 | 0.217 | 0.994 | 0.985 | 1.003 |
| PERK\_multiply | 0.001 | 0.002 | 0.130 | 1 | 0.719 | 1.001 | 0.997 | 1.004 |
| Histologic grade | 0.595 | 0.391 | 2.319 | 1 | 0.128 | 1.813 | 0.843 | 3.899 |
| pT | 0.625 | 0.302 | 4.288 | 1 | 0.038 | 1.868 | 1.034 | 3.375 |
| LN\_metastasis | 0.578 | 0.430 | 1.807 | 1 | 0.179 | 1.783 | 0.767 | 4.145 |
| FOXO3\_cyto\_multiply | -0.003 | 0.004 | 0.763 | 1 | 0.383 | 0.997 | 0.989 | 1.004 |
