# Supplementary material for: Regulation of PERK expression by FOXO3: a vulnerability of drug-resistant cancer cells
Source: Oncogene. 2019 Jul 16;38(36):6382–98. doi: 10.1038/s41388-019-0890-7 (PMC6756075; doi:10.1038/s41388-019-0890-7)
Supplement: Supplementary file 10 — Supplementary Figure Legends [file 41388_2019_890_MOESM10_ESM.docx]

**Supplementary Figure Legends**

**Supplementary Figure S1**

**Correlations between PERK expression and FOXO3 activity (expression/dephosphorylation) in MCF-7 and MCF-7-Tax^R^ cells in response to paclitaxel**

The paclitaxel sensitive (MCF-7) and resistant MCF-7-Tax^R^ cells were either left untreated or treated with 10 nM paclitaxel for the times mentioned. Whole-cell protein lysates were then analysed by western blotting using the antibodies against the proteins indicated. Western blotting was performed to determine the protein expression levels of P-FOXO3 (T32) (95kDa), total FOXO3 (95kDa), FOXM1 (110kDa) and ER stress molecules including p-PERK (140 kDa) total PERK (140 kDa), P-eIF2a (38kDa), total eIF2a (38kDa), p27^Kip1^ (27kDa) and β-Tubulin (55kDa) used as a loading control. Molecular weight markers are shown. **B.** FOXO3, PERK, p27Kip1 mRNA expression after treatment with paclitaxel as determined by RT-qPCR. Representative RNA expression profiles of at least 3 independent experiments. Three technical repeats were conducted in one experiment, and the data were normalized to L19 and displayed as means ± S.E.M. The expression trends of mRNA species between MCF-7 and MCF-7-Tax^R^ cells are compared using 2-way ANOVA (Significant ***p < 0.001, for all mRNA species, respectively).

**Supplementary Figure S2**

**Protein levels of MCF-7 transfected with non-silencing (NCS), FOXO1 and FOXO3 siRNA pools, showing compensation between FOXO1 and FOXO3 expression. A.** Western blotting was performed to study the protein expression levels for P-FOXO3 (T32) (95kDa), total FOXO3 (85kDa), P-FOXO1 (T24) (79kDa), total FOXO1 (79kDa), p-PERK (140kDa) total PERK (140 kDa), P-eIF2a (38kDa), total eIF2a (38kDa) and β-Tubulin (55kDa) as loading control. **B.** Expression of FOXO3 mRNA was determined by RT-qPCR analysis using L19 as an internal control. Three technical repeats were conducted in one experiment, and the data were normalized to L19 and displayed as means ± SEMs.

**Supplementary Figure S3.**

**FOXO3 and PERK expression is downregulated and PERK activity upregulated in the drug resistant MCF-7-Epi^R^ and MCF-7-Tax^R^ .**

Western blotting was performed on MCF-7, MCF-7-Epi^R^ and MCF-7-Tax^R^ cells to investigate the protein expression levels for P-FOXO3 (T32) (95kDa), total FOXO3 (85kDa), P-PERK (140kDa), total PERK (140 kDa), P-eIF2a (38kDa), total eIF2a (38kDa) and β-Tubulin (55kDa) as loading control.

**Supplementary Figure S4.**

**MCF-7-Epi^R^ and MCF-7-Tax^R^ with and without FOXO3 ectopic expression in response to PERK inhibitor GSK2606414 treatment.** MCF-7-Epi^R^ and MCF-7-Tax^R^ cells transfected with empty vector or FOXO3 expression vector were untreated or treated with PERK inhibitor GSK2606414. The transfected cells were analysed for their sensitivity to GSK2606414 by clonogenic assays. After 48 h of incubation with the drugs, cells were culture in fresh media, grown for around 14 days and stained with crystal violet. The clonogenic results of the cells were normalised to that of the cells without FOXO3 ectopic expression and GSK2606416. Data are representative of 3 independent experiments. Data represent means ± SEM. Significant **P* < 0.05, ****P* < 0.001.

**Supplementary Figure S5.**

**MCF-7 with and without FOXO3 ectopic expression in response to epirubicin treatment.** MCF-7 cells transfected with empty vector or FOXO3 expression vector were untreated or treated with 1 µM of epirubicin over a period 24 h. **A.** Expression of P-FOXO3 (T32) (95kDa), total FOXO3 (95kDa), P-PERK (140kDa) total PERK (140 kDa), P-eIF2a (38kDa), total eIF2a (38kDa) was determined by Western blot analysis using β-tubulin as loading control. **B.** Cell proliferation was assessed at 48, and 72 h after epirubicin treatment using SRB assay. The experiment was carried out in triplicates and data presented as means of the percentage of untreated control. Significant ***P* < 0.01.

**Supplementary Figure S6.**

**MCF-7 with and without FOXO3 ectopic expression in response to PERK inhibitor GSK2606414 treatment.** MCF-7 cells transfected with empty vector or FOXO3 expression vector were untreated or treated with 1 µM of the PERK inhibitor GSK2606414. **A.** Expression of P-FOXO3 (T32) (95kDa), total FOXO3 (85kDa), P-PERK (140kDa) total PERK (140 kDa), P-eIF2a (38kDa), total eIF2a (38kDa) was determined by Western blot analysis using β-tubulin as loading control. **B.** Expression of FOXO3 and PERK mRNA was determined by RT-qPCR analysis using L19 as an internal control. **C.** The transfected MCF-7 cells were analysed for their sensitivity to GSK2606414 by clonogenic assays. After 48 h of incubation with the drugs, cells were culture in fresh media, grown for around 14 days and stained with crystal violet. Data are representative of 3 independent experiments. Data represent means ± SEM; not significant ns.

**Supplementary Figure S7.**

**PERK expression in WT and *Foxo^1/3/4^* ^-/-^  MEFs following reintroduction of FOXO3.**

Expression levels of PERK protein and mRNA were analysed by Western blotting and RT-qPCR in WT and *Foxo^1/3/4^* ^-/-^ MEFs after transfection with FOXO3 expression vector. Western blotting was performed to determine the protein expression levels for Foxo3 (85kDa), P-Perk (140kDa), Perk (140kDa), P-eIF2a (38kDa), eIF2a (38kDa), p27Kip1 (27kDa) and β-Tubulin (55kDa) (Left panel). The relative Perk expression levels were calculated by the ratio of Perk to tubulin expression (Top right panel). Perk mRNA levels were investigated by RT-qPCR, and the data were normalized with L19 RNA level and displayed as means ± SEM (n=3; 2 tailed t-test). Significance: not significant, ns. Representative RNA expression profiles of at least 3 independent experiments are shown (lower right panel).

**Supplementary Figure S8.**

**Multivariate analysis to validate the prognostic values of FOXO3 and PERK expression and the clinicopathological parameters in HER2+ patient samples**
